# Creating sparser prediction models of treatment outcome in depression: a proof-of-concept study using simultaneous feature selection and hyperparameter tuning

**DOI:** 10.1186/s12911-022-01926-2

**Published:** 2022-07-14

**Authors:** Nicolas Rost, Tanja M. Brückl, Nikolaos Koutsouleris, Elisabeth B. Binder, Bertram Müller-Myhsok

**Affiliations:** 1 Department of Translational Research in Psychiatry, Max Planck Institute of Psychiatry, Kraepelinstraße 2-10, 80804 Munich, Germany; 2 International Max Planck Research School for Translational Psychiatry, Munich, Germany; 3 Department of Psychiatry and Psychotherapy, Ludwig Maximilian University, Munich, Germany; 4 Max Planck Institute of Psychiatry, Munich, Germany; 5 Institute of Psychiatry, Psychology and Neuroscience, King’s College, London, UK; 6 Department of Health Data Science, University of Liverpool, Liverpool, UK

**Keywords:** Major depressive disorder, Treatment outcome, Predictive modeling, Feature selection, Precision psychiatry, Supervised learning

## Abstract

**Background:**

Predicting treatment outcome in major depressive disorder (MDD) remains an essential challenge for precision psychiatry. Clinical prediction models (CPMs) based on supervised machine learning have been a promising approach for this endeavor. However, only few CPMs have focused on model sparsity even though sparser models might facilitate the translation into clinical practice and lower the expenses of their application.

**Methods:**

In this study, we developed a predictive modeling pipeline that combines hyperparameter tuning and recursive feature elimination in a nested cross-validation framework. We applied this pipeline to a real-world clinical data set on MDD treatment response and to a second simulated data set using three different classification algorithms. Performance was evaluated by permutation testing and comparison to a reference pipeline without nested feature selection.

**Results:**

Across all models, the proposed pipeline led to sparser CPMs compared to the reference pipeline. Except for one comparison, the proposed pipeline resulted in equally or more accurate predictions. For MDD treatment response, balanced accuracy scores ranged between 61 and 71% when models were applied to hold-out validation data.

**Conclusions:**

The resulting models might be particularly interesting for clinical applications as they could reduce expenses for clinical institutions and stress for patients.

**Supplementary Information:**

The online version contains supplementary material available at 10.1186/s12911-022-01926-2.

## Background

Despite many efforts in psychiatric research, the question of which patient will respond to which treatment is still unanswered. Specifically for very heterogenous disorders, such as major depressive disorder (MDD), no reliable (bio-)markers have been uncovered yet and no validated tests are available that could match a patient to the treatment they would benefit from the most [1, 2]. Predicting how well patients will respond to medication in general would be an important improvement for psychiatric health care and a further step towards precision medicine in psychiatry. Given the complex pathogenesis of psychiatric disorders, including MDD, it is unlikely that a few single indicators will be sufficient to forecast a patient’s response to pharmacotherapy. Rather, it will be important to collect a variety of measurements and gather information from many potentially informative data modalities [2].

The need to combine information from many different sources is why prognostic multivariate clinical prediction models (CPMs) might be particularly important in psychiatry. CPMs, and precision psychiatry in general, are fueled by data: the more features (in terms of measured patient characteristics) are available, the higher the chances of finding predictive variables. And the more samples are available, the higher the chances to obtain robust and generalizable models. Most prediction models, including those targeting treatment outcome in MDD, use supervised machine leaning techniques in order to maximize predictive power and generalizability at the same time [3]. However, when there are more features than samples in the data, the risk of overfitting the model increases and its generalizability decreases. This is often the case for data sets from patient cohorts, especially when high-dimensional biological data, such as (epi-)genetics and brain imaging, are included [4].

With the increasing availability of large data sets and simultaneous advances in bioinformatics and computational power, several multivariate prognostic models for predicting treatment outcome have been developed. We will use research on MDD and treatment with antidepressant medication as an example here. In general, however, CPMs are relevant for any condition in which there is a need to combine a multitude of predictors because no sufficiently predictive single factors have been identified so far [5].

Chekroud et al. [6] used data from the Sequenced Treatment Alternatives to Relieve Depression (STAR*D) study [7] in order to train a supervised machine learning model that was able to predict patients’ responses to the selective serotonin reuptake inhibitor escitalopram across different clinical trials with accuracies of 60–65%. Before training the model, they reduced the set of predictors by applying an elastic net regularized logistic regression [8] and kept the 25 most predictive variables (out of 164 initial variables). Dinga et al. [9] created a CPM of MDD long-term outcome based on observational data from the Netherlands Study of Depression and Anxiety [10]. The model was trained on different data modalities and included feature selection via elastic net regularization as well. It was able to differentiate between 3 patient groups (remission, improving, and chronic) with balanced accuracies of 60–66%. While these studies identified the most predictive variables using an entirely data-driven approach, i.e. via regularization techniques, other studies selected their variables a priori based on findings from previous research. Iniesta et al. [11], for instance, entered into their predictive models only demographic and clinical information that had been associated with treatment outcome in prior studies. They tested four different combinations of predictors, from a comparably sparse set of 60 variables up to 125, in order to evaluate the additional value of certain subgroups of variables. The best performing model predicted response to escitalopram with an area under the receiver operating characteristics curve of 0.75. Similarly, Athreya et al. [12] focused on previously identified factors in form of pharmacogenetic markers from genome-wide association studies. In combination with depression symptom scores, these markers predicted treatment response with accuracies between 71% and 86%. When applied to validation data sets, however, the model performances decreased below statistical significance. Further prediction models of MDD treatment outcome have been summarized in systematic reviews and meta-analyses [13, 14].

In general, CPMs are aimed at being translated and applied in clinical settings. They should be based on patient data that physicians can easily assess during their daily routine and should not require a lot of additional time and costs [15]. Consequently, the input data the model needs to make a prediction should be as sparse and cost-effective as possible [16]. If two models perform equally well, the simpler model should be preferred and will also be more likely to succeed as a clinical application, especially when the more complex model requires expensive additional measures. However, the majority of CPMs have either been constructed on a fixed, a priori selected feature set [6, 11, 12, 17], or included feature selection only in form of intrinsic regularization techniques [9]. None of the applied methods have used any further feature selection technique incorporated into the training process in order to develop sparser models. While regularization can effectively remove uninformative features from the final model, it cannot guarantee that an alternative model built on even less features would not perform equally well or even better when applied to new data. Hence, it might be beneficial to include an additional data-driven feature selection into the optimization framework in order to not just tune the model’s hyperparameters but also the required input feature set.

Different feature selection methods exist that can be implemented into a predictive modeling pipeline. In general, apart from the abovementioned intrinsic feature selection, e.g., by adding regularization terms to a regression model, the two main selection methods are filters and wrappers [18]. Filter approaches use the relationship between features and target for selection by ranking features according to the strength of their association with the target variable. The top N features, where N is usually defined by a certain cut-off, are then retained for the predictive modeling while the remaining features are discarded. A disadvantage of this technique is that relations between the features are not considered. Wrapper approaches, on the other hand, use searching techniques to find the most informative set of features. They create many different subsets of the input features and then select on the best performing subset according to a performance metric. These approaches can be more comprehensive, but also more computationally expensive [18]. Apart from feature selection methods, other techniques for dimensionality reduction exist, often including feature transformation, such as principal component analysis or multidimensional scaling. An overview over feature reduction methods for supervised learning problems is presented in Table 1.

**Table 1:** Common feature reduction approaches for supervised machine learning

Method	Description	Examples	Evaluation
*Feature selection*			
Intrinsic/embedded methods	Feature selection is implemented into the learning algorithm and performed during training	Regularized regression models Decision trees	Computationally efficient Interconnected with learning algorithm No guarantee of optimal sparsity
Filter methods	Feature selection based on associations with target variable	Associations are calculated using, e.g., correlations or ANOVA; top N features (or N%) are retained for training	Computationally efficient Relations between features ignored Independent of learning algorithm
Wrapper methods	Selection of best performing subset of features	Recursive feature elimination Sequential forward selection	Extensive search over input feature space Interconnected with learning algorithm Consider relations between features Computationally expensive
*Feature transformation*			
Projection into lower-dimensional feature space	Data are transformed and new features are created	Principal component analysis Multidimensional scaling Matrix factorization	Further methods of dimensionality reduction Alternative approaches to feature selection

*ANOVA*, analysis of variance

In this study, we compared a standard predictive modeling pipeline, that is, a repeated cross validation (CV) framework, to the same pipeline with an additional wrapper method for feature selection, i.e., recursive feature elimination (RFE) nested within the CV. We investigated three commonly used classifiers applied to two different data sets: one real-world data set from an observational inpatient study on patients with MDD as well as one simulated data set with similar dimensions. Our research questions were threefold: First, does the combined hyperparameter tuning and feature selection approach lead to models with sparser feature sets than intrinsic feature selection alone? Second, are classification accuracies between the two pipelines comparable or does the additional feature selection lead to changes in model performance? Third, does permutation testing lead to accuracies around chance level and can thus confirm that there is no information leakage biasing the results?

## Material & methods

### Data sets

Two different data sets were included in our analyses. First, as a real-world clinical data set, we used data from the Munich Antidepressant Response Signature (MARS) project [19], a multicenter naturalistic inpatient study, in which patients diagnosed with a single depressive episode, recurrent depressive disorder, or bipolar disorder were observed during their hospitalization. Further information on the study protocol and exclusion criteria have been published elsewhere [19]. The MARS study was approved by the ethics committee of the Ludwig Maximilian University in Munich, Germany, and conducted according to the Declaration of Helsinki. For our analyses, clinical response after 6 weeks of treatment, defined by at least 50% symptom reduction on the 17-item Hamilton Rating Scale for Depression (HDRS-17) [20], was used as a binary target variable for the CPMs. Patient characteristics measured at baseline, i.e., within the first week after study inclusion, were eligible as features for the predictions. We limited the analysis to unipolar depression and excluded patients diagnosed with bipolar disorder as well as patients without HDRS-17 scores at week 6 and patients with at least 75% missing values across all baseline features. Data from the resulting 1022 patients were then randomly split into a training (80%, 817 patients) and validation set (20%, 205 patients). From initially 548 baseline features, we removed those with at least 30% missing values as well as strongly imbalanced binary variables (ratio of 95:5% or more extreme), resulting in a final number of 113 features. The final feature set included sociodemographic data as well as information on psychiatric symptom profiles, symptom severity, family history, history of MDD, and medication. An overview over all included clinical features is presented in Additional file 1: Table S1. A flow diagram of all preprocessing steps that led to the final sample and feature selection is depicted in Additional file 1: Fig. S1.

The second data set consisted of simulated data with similar characteristics. Using Python’s *scikit-learn* package, we generated 1000 samples with 2 target classes and 125 features, consisting of 25 informative, 50 redundant, and 50 uninformative variables. Similar to the clinical data, the samples were randomly split into 800 training and 200 validation samples.

### Predictive modeling pipelines

**Fig. 1:**
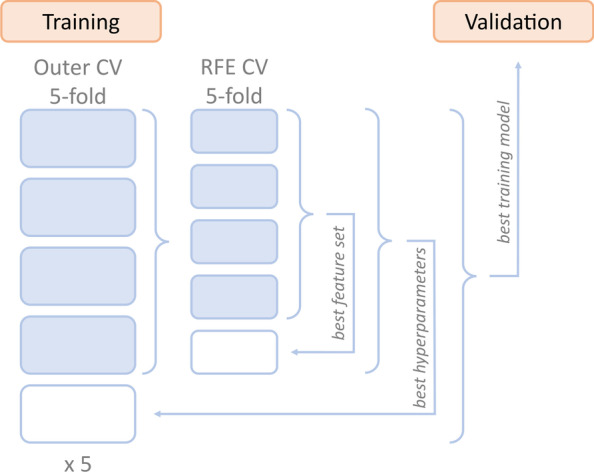
Supervised machine learning pipeline based on repeated nested cross-validation combining hyperparameter tuning and feature elimination. RFE, recursive feature elimination

All analyses were performed in Python (version 3.8.5) using the *scikit-learn* package (version 0.23.1) [21] and additional custom functions. The predictive modeling consisted of three different methods: (1) the proposed repeated nested CV with a simultaneous optimization of hyperparameters and best performing feature set; (2) a reference pipeline without the nested feature selection method; (3) 100 runs of the complete proposed pipeline from method (1) but with randomly permuted target variables. The proposed nested CV pipeline is additionally illustrated in Fig. 1. It entails a repeated (5 times) nested 5-by-5-fold CV, where the outer CV is used for hyperparameter tuning and the inner CV is used for RFE, implemented with *scikit-learn*’s *RFECV()* function. The goal of RFE is to select features by iteratively testing smaller feature sets. Initially, the model is trained on the entire feature set and the importance of each feature is extracted. Then, in a stepwise process, the feature with the lowest predictive power is gradually removed from the feature set until the best performing set of features is found. In our approach, the performance of the model is evaluated on a test set using CV. Therefore, in this framework, feature selection could happen both intrinsically, e.g., by the tuning of regularizing hyperparameters, and by the RFE. The final model was then defined by the on average best performing combination of hyperparameters and feature sets across all test folds. The second method was included as a reference to represent a common supervised machine learning pipeline. It consisted of a repeated (5 times) 5-fold CV used for hyperparameter tuning. Hence, it was identical to the proposed pipeline except for the nested RFE, and feature selection was only possible through intrinsic selection. The final model was defined by the on average best performing combination of hyperparameters across all folds. The third method was included as a permutation test for the proposed first pipeline in order to rule out the possibility of information leakage. It consisted of 100 runs of the complete nested CV pipeline but with randomly permuted target variables.

All three methods were applied to the two data sets using three different types of classifiers: an elastic-net regularized logistic regression (LR), a random forest classifier (RF), and a linear support vector classifier (SVC). Elastic-net regularized LR combines two different kinds of penalties (L1 or Lasso and L2 or Ridge) on the model which are commonly used to reduce complexity when the number of features is large [22]. This way, the risk of overfitting can be reduced by shrinking the feature coefficients and reducing multicollinearity. The ratio between the two penalties is usually tuned as a hyperparameter. RF is an ensemble learner that uses the results of a large number of decision trees to make the best possible classification. Single decision trees are uncorrelated and make individual decisions on its own. From the set of individual decisions, the RF provides a final decision [23]. Linear SVCs try to find optimal separation lines between the samples of different classes that can then be used to assign new samples to the correct class. These decision boundaries are chosen to maximize the distance between the data points of the classes so that future data points can be classified with the greatest possible confidence [24]. The three classifiers were selected in order to cover linear (all three classifiers) and non-linear (RF) associations of the features with the target variable and because they provide measures of importance (coefficients/weights) for each feature. Furthermore, they have frequently been used for various CPMs in psychiatry [6, 11, 25, 26]. Additional data preprocessing included k-nearest neighbors imputation of missing values [27] for all three classifiers and feature standardization for LR and SVC classification. Both steps were embedded into the (nested) CV, i.e., were created on the training folds and applied to the corresponding test fold of the CV loop. Hyperparameter tuning during model fitting was performed using Bayesian optimization [28]. After training, the resulting models were applied to the validation data set in order to get a final performance estimate. Crucially, the validation data set was completely left out of the training process and its CV loops. Such external validation on a hold-out data set is necessary to assess model performance independently of the training data on ‘new’ and ‘unseen’ data. Performance was primarily measured by Matthews correlation coefficient (MCC) [29] and the balanced accuracy score (BAC) [30]. Additionally, we extracted receiver operating characteristic curves and confusion matrices of all non-permuted classifiers. Since the MCC is a special form of the Pearson correlation coefficient, a value of 0 corresponds to chance level. For BAC scores, the chance level of a binary classifier is 0.5. MCC values from the permuted models across both data sets and all three classifiers were tested against their theoretical null distribution, that is, a t-distribution with n-2 degrees of freedom [31], using Kolmogorov–Smirnov tests. Statistical significance of the non-permuted models was tested using *p*-values derived from the same distribution. To compare the models with RFE to the models without RFE, we performed pairwise tests on the respective MCC values [32]. Further, for the non-permuted models, the importance of each feature was calculated by its permutation importance on the validation data, that is, by the average decrease in model performance when the feature was randomly permuted. The number of permutations for this procedure was set to 25.

### Availability of data and materials

Data from the MARS study as well as the corresponding preprocessed data set that was used for the analyses can be requested by contacting Dr. Tanja Brückl (brueckl@psych.mpg.de). The TRIPOD (Transparent Reporting of a multivariable prediction model for Individual Prognosis Or Diagnosis) [33] checklist for the present study is presented in Additional file 1: Table S2. Analysis scripts are available at https://doi.org/10.5281/zenodo.6759730.

## Results

In the clinical data set, 564 out of 1022 patients (55.19%) showed a clinical response, defined by at least 50% symptom reduction measured with the HRDS-17 sum score after 6 weeks of antidepressant treatment, whereas 458 patients (44.81%) did not respond. Hence, the outcome groups were slightly unequally large which is why the classifiers’ class weights were balanced. Demographic data and basic clinical information for training and validation set are presented in Table 2. In the simulated data set, the outcome groups were created to be balanced with 500 samples in group 1 and 500 samples in group 2.

**Table 2:** Basic patient characteristics of the clinical data set (MARS study)

	Training data (N = 817)	Validation data (N = 205)	Overall (N = 1,022)	p
*Gender*				
Female	431 (52.8%)	105 (51.2%)	536 (52.4%)	0.753
Male	386 (47.2%)	100 (48.8%)	486 (47.6%)	
*Age*				
Mean (SD)	47.4 (14.0)	47.1 (14.4)	47.3 (14.1)	0.790
[Min, Max]	[18.0, 85.0]	[18.0, 87.0]	[18.0, 87.0]	
*Diagnosis (ICD-10)*				
F32	289 (35.4%)	61 (29.8%)	350 (34.2%)	0.152
F33	528 (64.6%)	144 (70.2%)	672 (65.8%)	
*HDRS-17 baseline sum score*				
Mean (SD)	24.0 (5.6)	23.4 (5.5)	23.8 (5.6)	0.185
[Min, Max]	[12.0, 40.0]	[10.0, 39.0]	[10.0, 40.0]	
Missing	11 (1.3%)	4 (2.0%)	15 (1.5%)	
*HDRS-17 response*				
Yes	454 (55.6%)	110 (53.7%)	564 (55.2%)	0.679
No	363 (44.4%)	95 (46.3%)	458 (44.8%)	

Two sample t-tests were computed for continuous variables, Chi-squared tests were used for categorical variables to compare training and test data set

*HDRS-17*, 17-item version of the hamilton rating scale for depression; *ICD-10*, international classification of diseases [34]

### Model performances

**Fig. 2:**
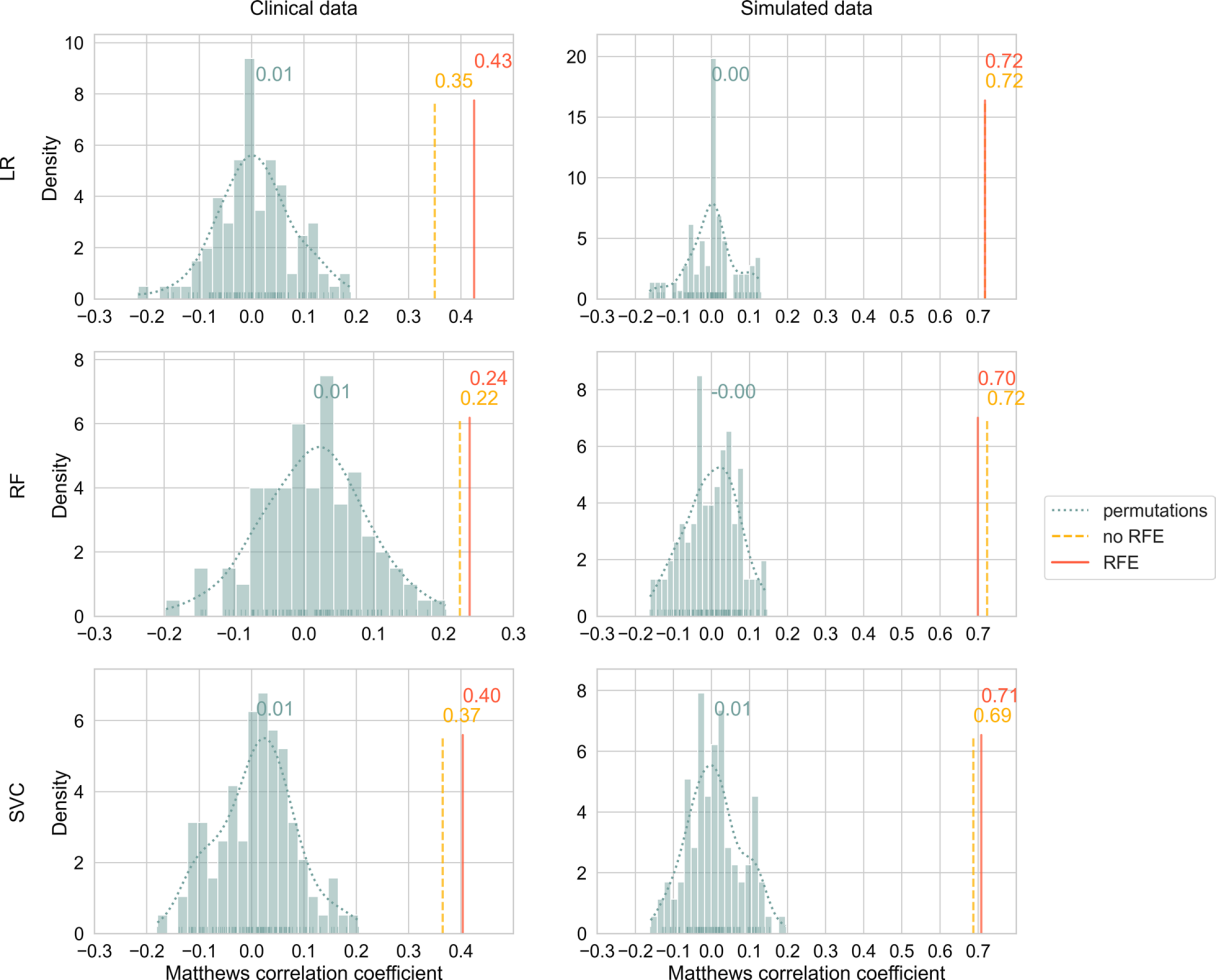
Model performances for the three classifiers and the two data sets on the validation data. Matthews correlation coefficients are shown for the 100 permutations (annotations correspond to the respective means) as well as for the models with and without RFE. LR, logistic regression; RF, random forest classifier; RFE, recursive feature elimination; SVC, support vector classifier

Classification performances of the non-permuted models (with and without RFE) for the clinical data ranged from MCC values of 0.22 up to 0.43 (BAC scores: 0.61–0.71). For the simulated data, MCCs between 0.69 and 0.72 were observed (BAC scores: 0.84–0.86). Figure 2 shows the MCCs of the validation data for all computed models (for corresponding BAC scores, see Additional file 1: Fig. S2). Model performances of the non-permuted models are represented by vertical bars. Results from the 100 permutations are indicated by histograms, superimposed density curves and the respective average performance. Across all six comparisons, performances of the modeling pipeline with RFE and the pipeline without RFE were relatively similar. No significant differences were observed between the two pipelines (see Table 3). Interestingly, in four of the six cases, the models with RFE loop resulted in better predictions on the hold-out validation set than the models without RFE (all three classifiers on clinical data and SVC on simulated data). In one of the cases (LR on simulated data), MCCs and BAC scores were equal up to the second decimal place, and in one case (RF on simulated data), the model without RFE was superior. All non-permuted models both with and without RFE performed significantly better than chance, indicated by the *p*-values of the MCCs (all *p* < 0.01, see Additional file 1: Table S3). To further characterize the modeling results, we included the receiver operating characteristic curves and the corresponding areas under the curves in Additional file 1: Fig. S3. Confusion matrices and additional performance metrics, such as sensitivity and specificity of the classifiers, are represented in Additional file 1: Table S4.

**Table 3:** Pairwise statistical significance tests between model performances (MCC values) of the models with and without RFE on the validation data

	MCC		z	*p*
	RFE	No RFE		
*Clinical data (N* = *205)*				
LR	0.425	0.350	0.888	0.375
RF	0.237	0.224	0.138	0.890
SVC	0.403	0.365	0.448	0.654
*Simulated data (N* = *200)*				
LR	0.718	0.719	− 0.021	0.984
RF	0.700	0.724	− 0.483	0.629
SVC	0.709	0.688	0.407	0.684

*LR*, logistic regression; *MCC*, Matthews correlation coefficient; *RF*, random forest classifier; *RFE*, recursive feature elimination; *SVC*, support vector classifier

When the target class labels in the RFE pipeline were randomly permuted 100 times, the resulting performance metrics became distributed around their chance levels as expected (0 for MCC and 0.5 for BAC, respectively). For MCC values, Kolmogorov–Smirnov tests showed no significant deviations from the theoretical null distribution (all *p* > 0.05, see Additional file 1: Table S5). These results suggested no unintended information leakage from training to validation data. Quantile–quantile plots of empirical and theoretical MCC distributions are presented in Additional file 1: Fig. S4. None of the permutation runs led to better model performances than the corresponding non-permuted models (see Fig. 2).

### Number of selected features

**Fig. 3:**
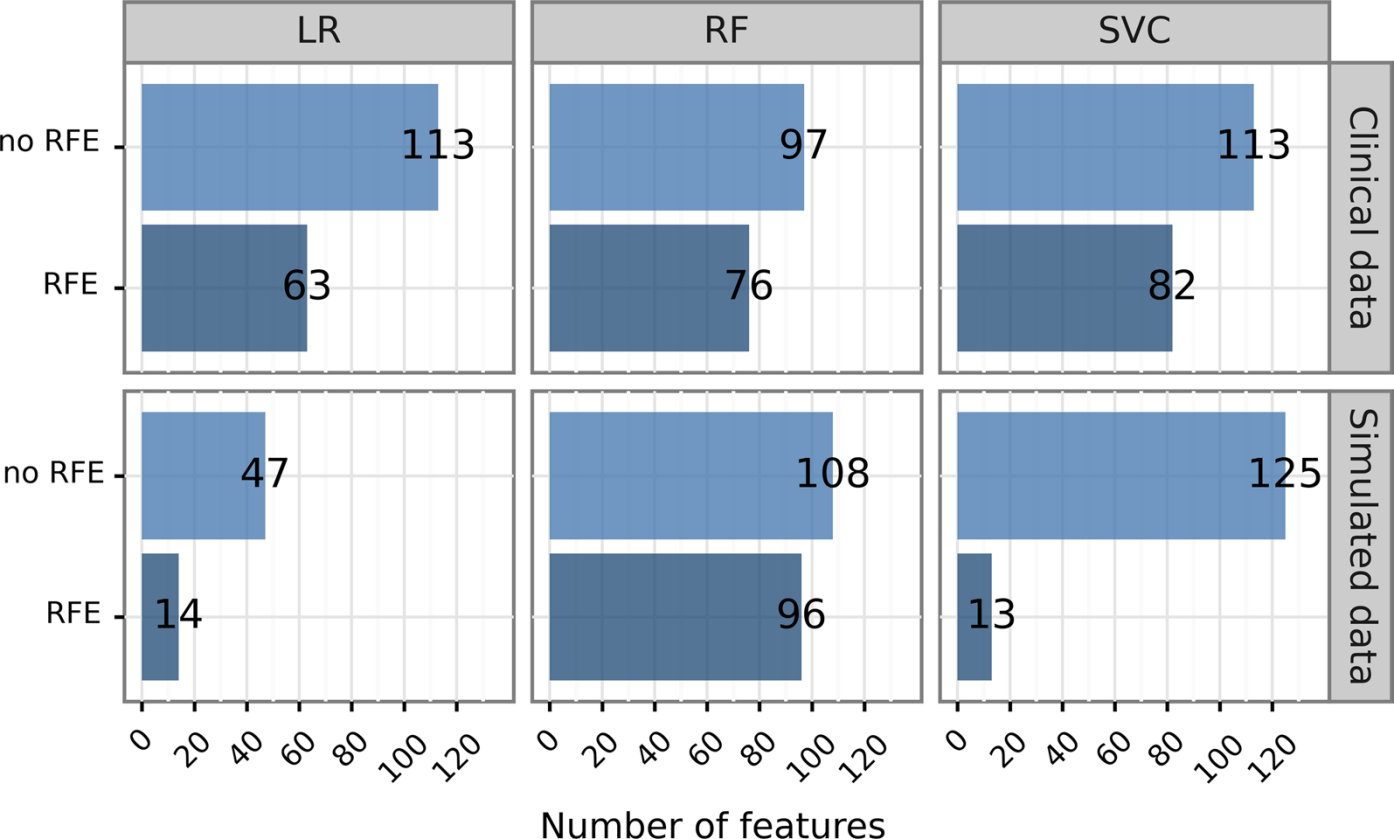
Number of selected features for the non-permuted models. Across both data sets and all three classifiers, the nested cross-validation pipeline with RFE (lower rows) resulted in sparser models requiring less features than the reference method without RFE (upper rows). LR, logistic regression; RF, random forest classifier; RFE, recursive feature elimination; SVC, support vector classifier

Overall, the RFE models resulted in sparser features sets than the models without RFE. Figure 3 shows the final numbers of features required by the models after intrinsic feature selection and selection via RFE. Across all six comparisons, the final models from the nested CV pipeline with RFE required less features than the equivalent models from the single CV pipeline without RFE. While for RFs, the RFE pipeline resulted in models requiring 76 and 96 features for the clinical and the simulated data, respectively, the models without RFE yielded 97 and 108 features with non-zero coefficients. Even stronger differences were obtained from the LR classifiers with differences of 50 features (clinical data) and 33 features (simulated data), and from the SVC models with differences of 31 features (clinical data) and 112 features (simulated data). Note that the pipeline without RFE could still lead to non-zero feature coefficients via intrinsic feature selection.

**Fig. 4:**
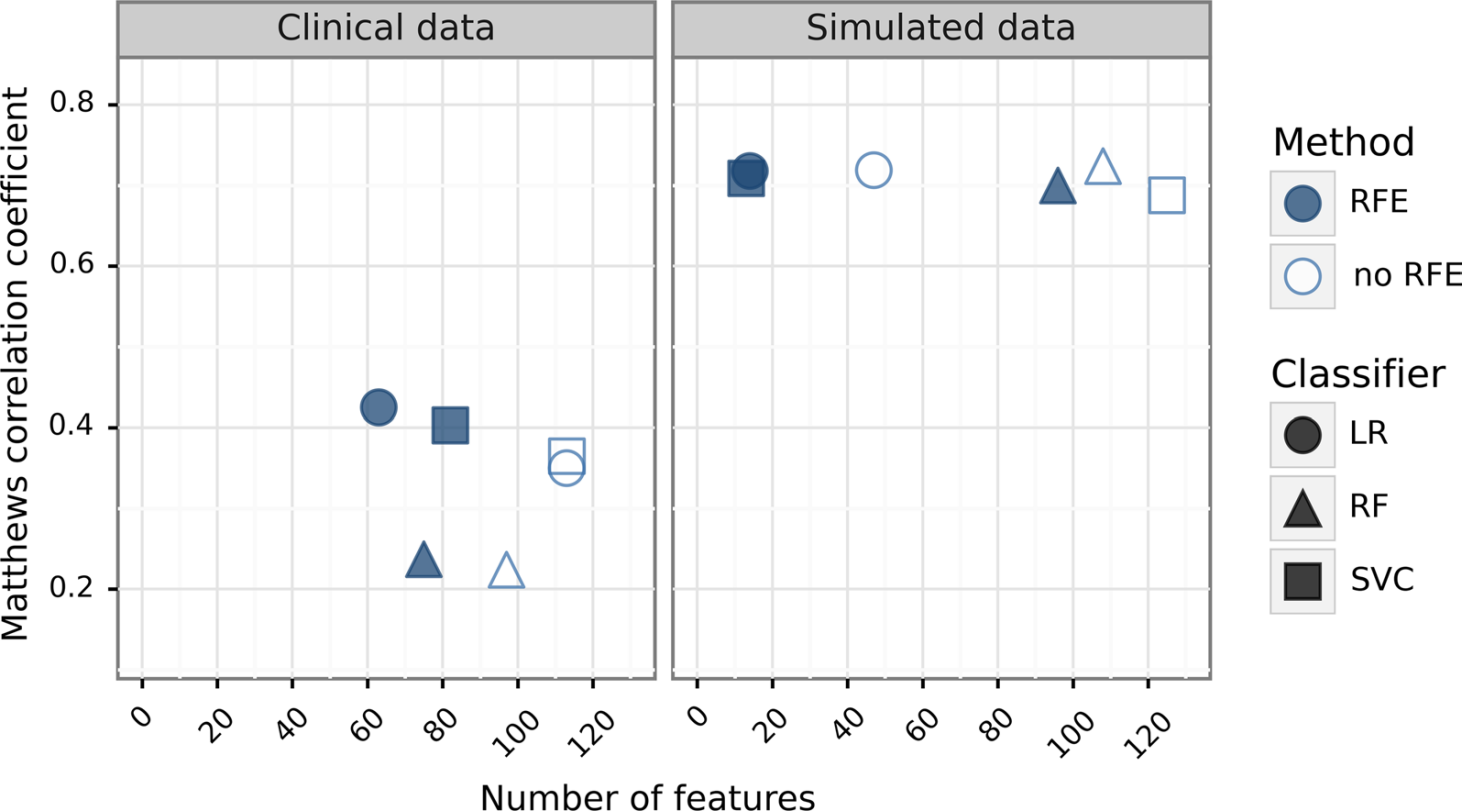
Number of selected features for the non-permuted models plotted against the corresponding model performances. The left plot represents the values for the clinical data set, the right plot for the simulated data set. The different shapes indicate the three classifiers. LR, logistic regression; RF, random forest classifier; RFE, recursive feature elimination; SVC, support vector classifier

Figure 4 provides a combined overview over the main results by simultaneously depicting model performances (indicated by MCC on the y-axis) and numbers of selected features (on the x-axis) of all non-permuted models. Overall, the nested CV pipeline with RFE seemed to outperform the reference pipeline without RFE as it resulted on average in better performing models while also requiring less input features.

### Clinical predictors of MDD treatment response

**Fig. 5:**
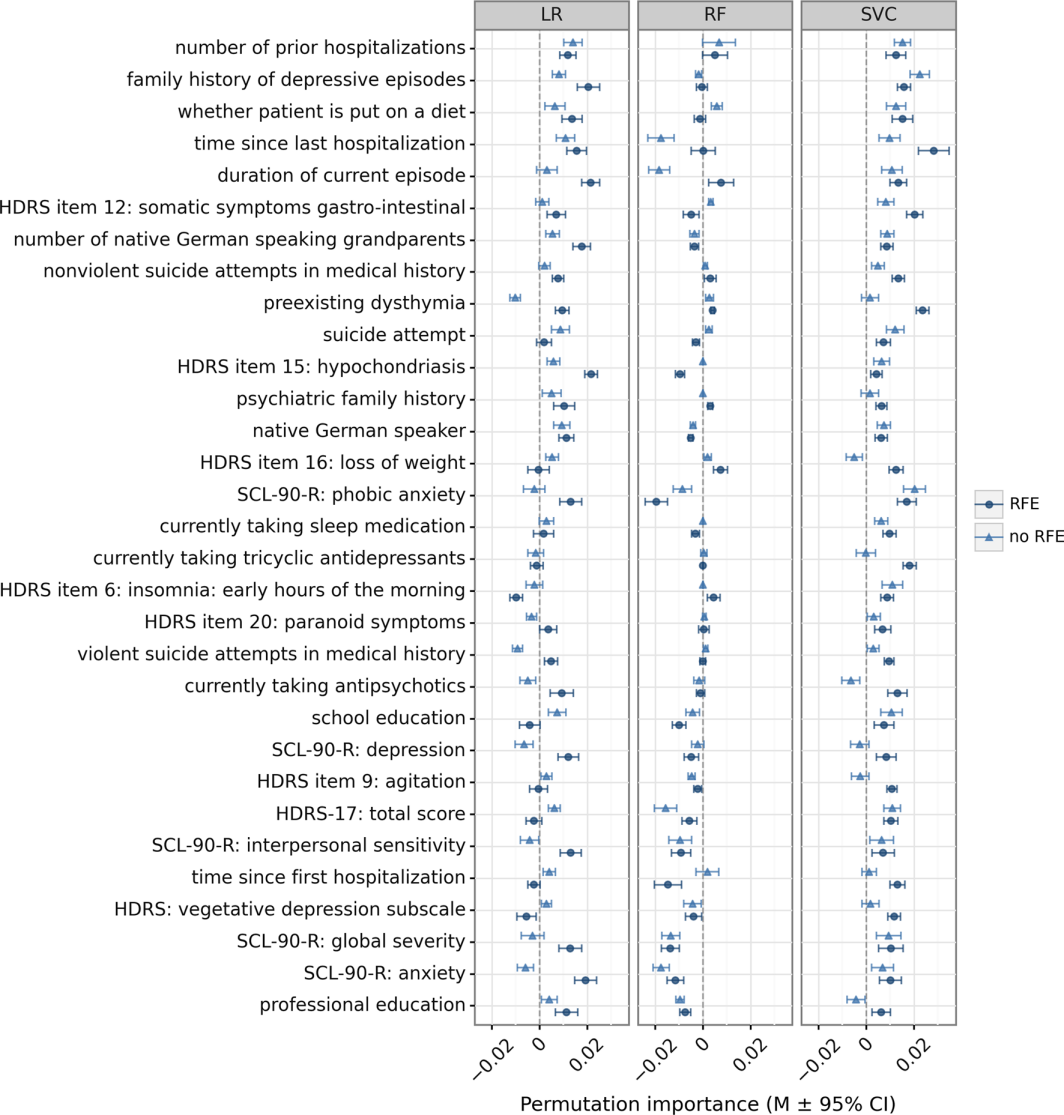
Permutation importance from 25 permutations for the most informative clinical features, grouped by classifier and models with and without RFE. Only features that were selected by all 6 clinical models and showed a positive mean importance score (averaged over all 6 models) are presented. The scores show the average decrease in model performance on the test data when a feature was randomly permuted. Error bars represent 95% confidence intervals. LR, logistic regression; RF, random forest classifier; RFE, recursive feature elimination; SVC, support vector classifier

For the clinical data set, we were additionally interested in the most important predictors of MDD treatment response. Therefore, the permutation importance for each feature in each model was calculated using 25 permutations applied to the validation data set. The most informative features and their corresponding importance values, sorted by their importance (averaged over the three classifiers and the two pipelines), are illustrated in Fig. 5. The most informative features included information on the course of the disorder (e.g., number of prior hospitalizations, time since last hospitalization, duration of current episode), family history (of psychiatric disorders and MDD specifically) as well as symptom profiles and severity (e.g., various item scores from the HDRS and the Symptom Check-List-90-R [SCL90-R]) [35]. While several features showed rather consistent importance values (e.g., number of prior hospitalizations, nonviolent suicide attempts in medical history, psychiatric family history), regardless of which classifier or which pipeline was applied, other features varied in their permutation importance depending on the model that was used (e.g., preexisting dysthymia, SCL-90-R phobic anxiety, HDRS-17: total score). Note that negative importance values indicate that a feature was non-informative for a model but shuffling this feature led to a better model performance by chance.

A complete overview over the importance values of all features in alphabetical order is included in Additional file 1: Fig. S5. A more detailed description of the complete clinical feature set in is given in Additional file 1: Table S1. Corresponding feature importance values for the simulated data set are presented in Additional file 1: Fig. S6 (top predictors sorted by importance) and Additional file 1: Fig. S7 (complete feature set).

## Discussion

In the present study, we tested whether a supervised machine learning pipeline that combined hyperparameter tuning and RFE in a repeated nested CV setup can lead to sparser but similarly accurate binary classification models than a default pipeline with only one CV loop for hyperparameter tuning. For this investigation, we used three different kinds of classification algorithms applied to two different data sets, one real-world data set on MDD treatment outcome and one simulated data set with similar dimensions. Our results showed that (1) the additional RFE loop led to sparser models that required less features for the classification; (2) although not statistically significant, the pipeline with RFE yielded equally well or better performing models on the validation data set in five of six cases; and (3) permutation tests suggested no unintended information leakage in the pipeline with RFE. Furthermore, all non-permuted models performed significantly better than chance, indicated by *p*-values < 0.01.

The results from the present study might be particularly relevant for classification tasks in clinical research. Clinical patient data sets are often based on comparably expensive measurements and sparser models requiring less features might not only decrease costs for clinical institutions but also stress for patients. Especially when expensive biological measures (e.g., brain imaging, -omics data) that need a lot of laboratory or computational capacities are included in data sets, it might be important to be rather strict on the inclusion of features into a predictive model. Measures that are not contributing strongly to the prediction should be omitted when there is a sparser model performing equally well or even better [16]. By using the pipeline proposed here, feature selection, hyperparameter tuning and model fitting can be performed in one nested data-driven optimization process. Hence, this approach does not require any prior theory-driven feature selection but automatically selects the best performing feature set for each of the tested combinations of hyperparameters. Measurement time and costs can be reduced when applying such a reduced, sparser model in clinical practice. Sparser models also help to increase data quality because patients have to fill in less questionnaires which reduces respondent fatigue. In our analyses, the additional RFE loop reduced the number of features required by the final model by 12 features in the least extreme case (RF on simulated data) and 112 features in the most extreme case (SVC on simulated data). With respect to the MDD data set, features containing information on the patient’s marital status, their gender, the origin of their grandparents and specific medication, for instance, were removed by the RFE across all three classifiers but had mostly non-zero feature coefficients in models created by the pipeline without RFE. By omitting these features, future applications of the model would require less information from the patients and could thus save time and efforts. While we have focused on RFE as a feature selection technique here, other filter or wrapper approaches might be similarly appropriate in general. In previous studies, different filter techniques have been successfully used for spam detection [36, 37], for instance, but have also been applied to biological human data [38, 39].

With respect to absolute performance of the predictive models, the observed performance values for the clinical data were within the expected range. The obtained MCCs of 0.22–0.43 and BAC scores of 0.61–0.71 were comparable to results from similar prior studies [13, 14]. Such classification accuracies of approximately 60–70% are far from ideal but might still be clinically relevant [40] and could provide support for clinicians in their treatment decisions. Our results underline that predicting antidepressant treatment outcome is a difficult and still unsolved endeavor, especially when the data set is as heterogenous as in our case. Since the MARS project was designed to be a naturalistic observational inpatient study, it included patients from various age groups with diverse symptom profiles and medical histories as well as different pharmacological treatments. On the other hand, it represents quite a realistic picture of the broad clinical spectrum of MDD. Regarding the simulated data (MCC: 0.69–0.72; BAC: 0.84–0.86), better performances compared to the clinical data were expected because 25 features were explicitly created to be informative for the target variable. The congruency of the main results across the two data sets highlights that the differences between the two pipelines do not depend on the overall informativeness of the features and might generalize to other data sets as well.

In addition to ‘traditional’ supervised machine learning algorithms, such as the classifiers applied in this study, deep learning in the sense of deep neural networks is becoming increasingly common in psychiatric research. So far, however, applications have rather focused on diagnosis than on prognosis or personalization of treatment [41]. A reason might be that deep learning usually requires large sample sizes and has an increased risk of overfitting due to the number of parameters fitted, especially in relatively small sample sizes that are common in psychiatric clinical trials [3, 42]. In addition, deep neural networks were shown to be not generally superior to other classifiers on many classification tasks [43–47], but come with comparatively high computational costs. However, for more complex features, such as brain imaging, time-series, or sensor-based data, prognostic research in psychiatry might benefit from deep learning [41, 42, 48]. There is also growing evidence that deep neural networks might be particularly useful for integration of multimodal data, e.g., from studies on stress detection [49] and diagnosing MDD [50] and Alzheimer’s disease [51–53].

With respect to treatment outcome, we selected a reduction of ≥ 50% on a symptom scale sum score after 6 weeks of treatment as the target variable for the clinical data set because it represents one of the most widely used definitions of treatment outcome in MDD research. Recently, more and more critique has come up on MDD measurement in general [54] and on symptom scale sum score-based outcome definitions in particular (for a review, see [55], for instance). The definition of response used here represents an artificial dichotomization of an ordinal scale and is therefore associated with loss of information [56]. While most MDD outcome classification models have aimed at such binary outcome definitions based on cut-off values [13, 14], others have used unsupervised learning to generate data-driven outcome classes beforehand [25]. So far, however, there is no evidence for the superiority of one outcome definition over another in terms of predictability.

Our study shows some limitations. First, our pipeline can only be applied to classification algorithms which provide some kind of feature coefficients, at least in the version of *scikit-learn* (0.23.1) that was used in the present study. SVCs with non-linear kernels, for instance, were not included in our analyses as they do not return feature coefficients required by the RFE. However, the applied classifiers represent a selection of commonly used classifiers for CPMs of MDD treatment outcome [14]. Second, it remains unclear how well our results generalize to data sets with very different dimensions, i.e., different sample-to-feature ratios. It is possible that data sets with significantly more or less features compared to the number of samples might profit less from the nested pipeline with RFE. Still, we tested our pipeline both on real and simulated data with dimensions that are representative of many psychiatric patient cohorts and corresponding CPM studies (e.g., [6, 9, 25]). Third, the proposed pipeline with nested RFE is computationally expensive compared to a single CV pipeline or a nested CV without RFE. Hence, we restricted our analyses to 100 permutation runs even though a larger number of permutations might have resulted in a more precise empirical null distribution. In future applications, it might be worth to evaluate first if the benefits of a sparser CPM would outweigh the additional computational expenses needed during model development.

## Conclusions

In conclusion, our nested supervised machine learning pipeline with simultaneous hyperparameter tuning and feature selection could lead to sparser CPMs without losses in accuracy. This approach might be particularly beneficial in scenarios in which a literature-based a priori feature selection is not possible, e.g., due to lack of evidence or, in contrast, due to a large number of potentially useful predictors, as observed in MDD, for instance [57]. If measurements that come with certain expenses are involved, sparser models could reduce both costs for users (e.g., clinical institutions) and stress for patients resulting in better data quality.

## Supplementary Information

**Additional file 1**: **Table S1**. Baseline features used for predictive modeling in the clinical data set in alphabetical order. **Fig. S1**. Preprocessing workflow of samples and features from the clinical data set. **Table S2**. TRIPOD Checklist for Prediction Model Development and Validation.** Fig. S2**. Balanced accuracy scores for the three classifiers and the two data sets on the validation data. **Table S3**. Matthews correlation coefficients and corresponding *p*-values for non-permuted models.** Fig. S3**. Receiver operating characteristic curves and corresponding AUC values for all non-permuted models (with and without RFE) across the three classifiers and the two data sets on the validation data. **Table S4**. Confusion matrices and derived performance metrics including 95% confidence intervals for all non-permuted models on the validation data. **Table S5**. Results from Kolmogorov-Smirnov tests comparing the empirical MCC distributions of the permutation runs to the theoretical null distribution. **Fig. S4**. Quantile-quantile plots for the 100 permutation runs of each classifier and data set. **Fig S5**. Permutation importance from 25 permutations for all 113 clinical features, ordered alphabetically and grouped by classifier and model. **Fig. S6**. Permutation importance from 25 permutations for the most informative features from the simulated data set, grouped by classifier and models with and without RFE.. **Fig. S7**. Permutation importance from 25 permutations for all 125 features from the simulated data set, ordered by number and grouped by classifier and model (with and without RFE).

## Data Availability

Data from the MARS study as well as the corresponding preprocessed data set that was used for the analyses can be requested by contacting Dr. Tanja Brückl (brueckl@psych.mpg.de).. Analysis scripts are available at https://doi.org/10.5281/zenodo.6759730.

## Abbreviations

BAC: Balanced accuracy
CPM: Clinical prediction model
CV: Cross-validation
LR: Logistic regression
MARS: Munich antidepressant response signature
MCC: Matthews correlation coefficient
MDD: Major depressive disorder
RF: Random forest classifier
RFE: Recursive feature elimination
STAR*D: Sequenced treatment alternatives to relieve depression
SVC: Support vector classifier
