# Supplementary material for: Creating sparser prediction models of treatment outcome in depression: a proof-of-concept study using simultaneous feature selection and hyperparameter tuning
Source: BMC Med Inform Decis Mak. 2022 Jul 14;22:181. doi: 10.1186/s12911-022-01926-2 (PMC9284749; doi:10.1186/s12911-022-01926-2)
Supplement: Supplementary file 1 — Additional file 1: Table S1. Baseline features used for predictive modeling in the clinical data set in alphabetical order. Fig. S1. Preprocessing workflow of samples and features from the clinical data set. Table S2. TRIPOD Checklist for Prediction Model Development and Validation. Fig. S2. Balanced accuracy scores for the three classifiers and the two data sets on the validation data. Table S3. Matthews correlation coefficients and corresponding p-values for non-permuted models. Fig. S3. Receiver operating characteristic curves and corresponding AUC values for all non-permuted models (with and without RFE) across the three classifiers and the two data sets on the validation data. Table S4. Confusion matrices and derived performance metrics including 95% confidence intervals for all non-permuted models on the validation data. Table S5. Results from Kolmogorov-Smirnov tests comparing the empirical MCC distributions of the permutation runs to the theoretical null distribution. Fig. S4. Quantile-quantile plots for the 100 permutation runs of each classifier and data set. Fig S5. Permutation importance from 25 permutations for all 113 clinical features, ordered alphabetically and grouped by classifier and model. Fig. S6. Permutation importance from 25 permutations for the most informative features from the simulated data set, grouped by classifier and models with and without RFE.. Fig. S7. Permutation importance from 25 permutations for all 125 features from the simulated data set, ordered by number and grouped by classifier and model (with and without RFE). [file 12911_2022_1926_MOESM1_ESM.doc]

**Supplementary Material**

**Rost, Brückl, Koutsouleris, Binder, & Müller-Myhsok. Creating sparser prediction models of treatment outcome in depression: A proof-of-concept study using simultaneous feature selection and hyperparameter tuning**

**Table of Contents**

[Figures 3](#__RefHeading___Toc107410626)

[Supplementary Figure 1. Preprocessing workflow of samples and features from the clinical data set (MARS project). 3](#__RefHeading___Toc107410627)

[Supplementary Figure 2. Model performances for the three classifiers and the two data sets, indicated by balanced accuracy scores. The scores are shown for the 100 permutations (annotations correspond to the respective means) as well as for the models with and without RFE. 4](#__RefHeading___Toc107410628)

[Supplementary Figure 3. Receiver operating characteristic curves and corresponding AUC values for all non-permuted models (with and without RFE) across the three classifiers and the two data sets. 5](#__RefHeading___Toc107410629)

[Supplementary Figure 4. Quantile-quantile plots for the 100 permutation runs of each classifier and data set. Empirical distributions of the MCC values on the y-axis are plotted against the theoretical distribution (t(n-2)-distribution) on the x-axis. The dotted line represents the identity function. 6](#__RefHeading___Toc107410630)

[Supplementary Figure 5. Permutation importance from 25 permutations for all 113 clinical features, ordered alphabetically and grouped by classifier and model (with and without RFE). The scores show the average decrease in model performance on the validation data when a feature was randomly permuted. Error bars represent 95% confidence intervals. Missing values indicate that a feature was removed by the RFE. 7](#__RefHeading___Toc107410631)

[Supplementary Figure 6. Permutation importance from 25 permutations for the most informative features from the simulated data set, grouped by classifier and models with and without RFE. Only features that were selected by all 6 models and showed a positive mean importance score (averaged over all 6 models) are presented. The scores show the average decrease in model performance on the validation data when a feature was randomly permuted. Error bars represent 95% confidence intervals. 8](#__RefHeading___Toc107410632)

[Supplementary Figure 7. Permutation importance from 25 permutations for all 125 features from the simulated data set, ordered by number and grouped by classifier and model (with and without RFE). The scores show the average decrease in model performance on the validation data when a feature was randomly permuted. Error bars represent 95% confidence intervals. Missing values indicate that a feature was removed by the RFE. 9](#__RefHeading___Toc107410633)

[Tables 10](#__RefHeading___Toc107410634)

[Supplementary Table 1. Baseline features used for predictive modeling in the clinical data set in alphabetical order. After preprocessing, 113 features remained. The table below provides further information on the variables and their distributions. 10](#__RefHeading___Toc107410635)

[Supplementary Table 2. TRIPOD Checklist for Prediction Model Development and Validation (8) 16](#__RefHeading___Toc107410636)

[Supplementary Table 3. Matthews correlation coefficients and corresponding p-values for non-permuted models (with and without recursive feature elimination). P-values were derived from the theoretical null distribution (t(n-2)-distribution). 18](#__RefHeading___Toc107410637)

[Supplementary Table 4. Confusion matrices and derived performance metrics including 95% confidence intervals for all non-permuted models on the validation data. 19](#__RefHeading___Toc107410638)

[Supplementary Table 5. Results from Kolmogorov-Smirnov tests comparing the empirical MCC distributions of the permutation runs to the theoretical null distribution (t(n-2)-distribution). 21](#__RefHeading___Toc107410639)

[References 22](#__RefHeading___Toc107410640)

# Figures

### Fig. S1. Preprocessing workflow of samples and features from the clinical data set (MARS project).

***
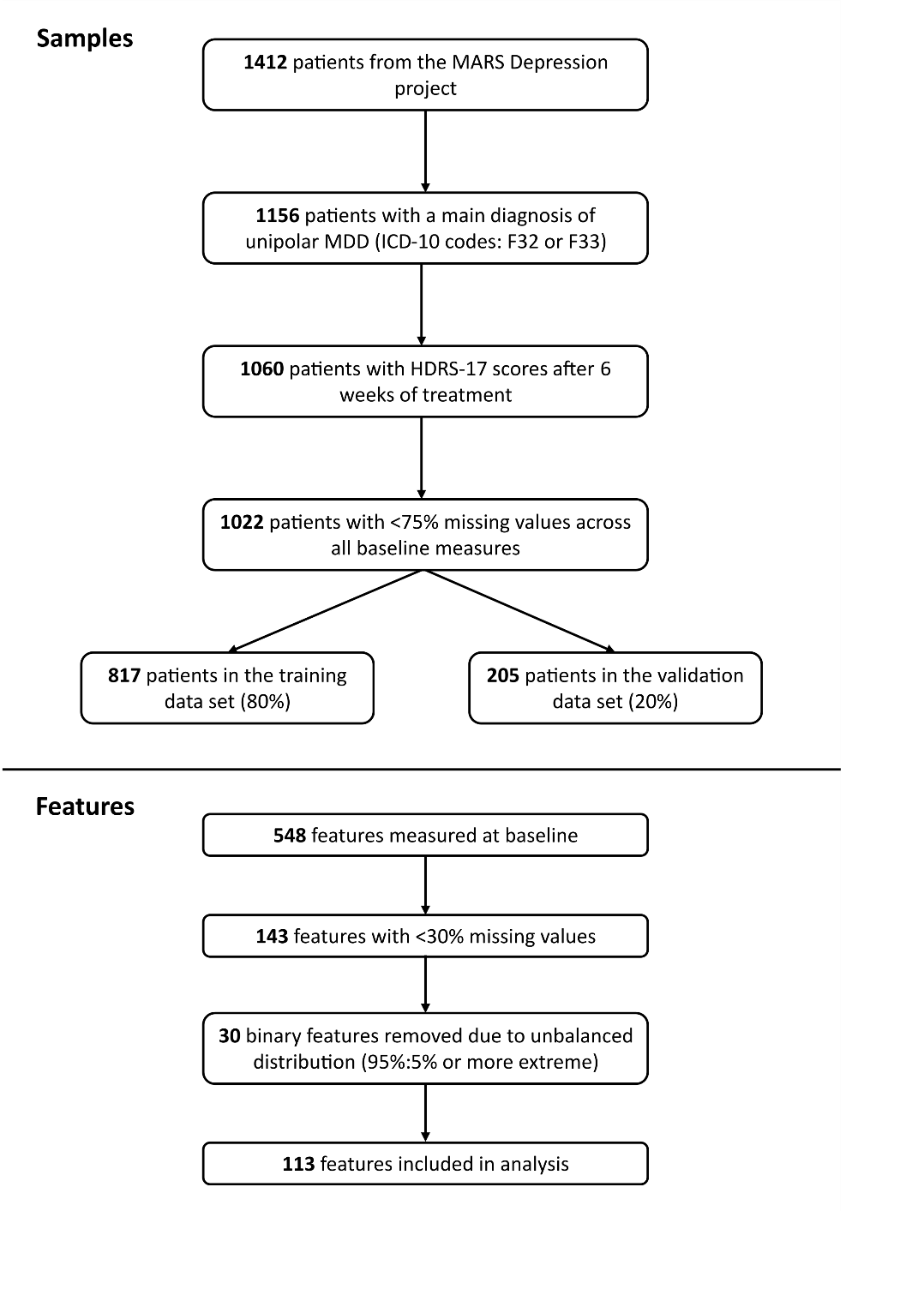
***

HDRS-17, 17-item version of the Hamilton Rating Scale for Depression (1); ICD-10, International Classification of Diseases (2); MARS, Munich Antidepressant Response Signature (3); MDD, Major Depressive Disorder.

### Fig. S2. Model performances for the three classifiers and the two data sets on the validation data, indicated by balanced accuracy scores. The scores are shown for the 100 permutations (annotations correspond to the respective means) as well as for the models with and without RFE.


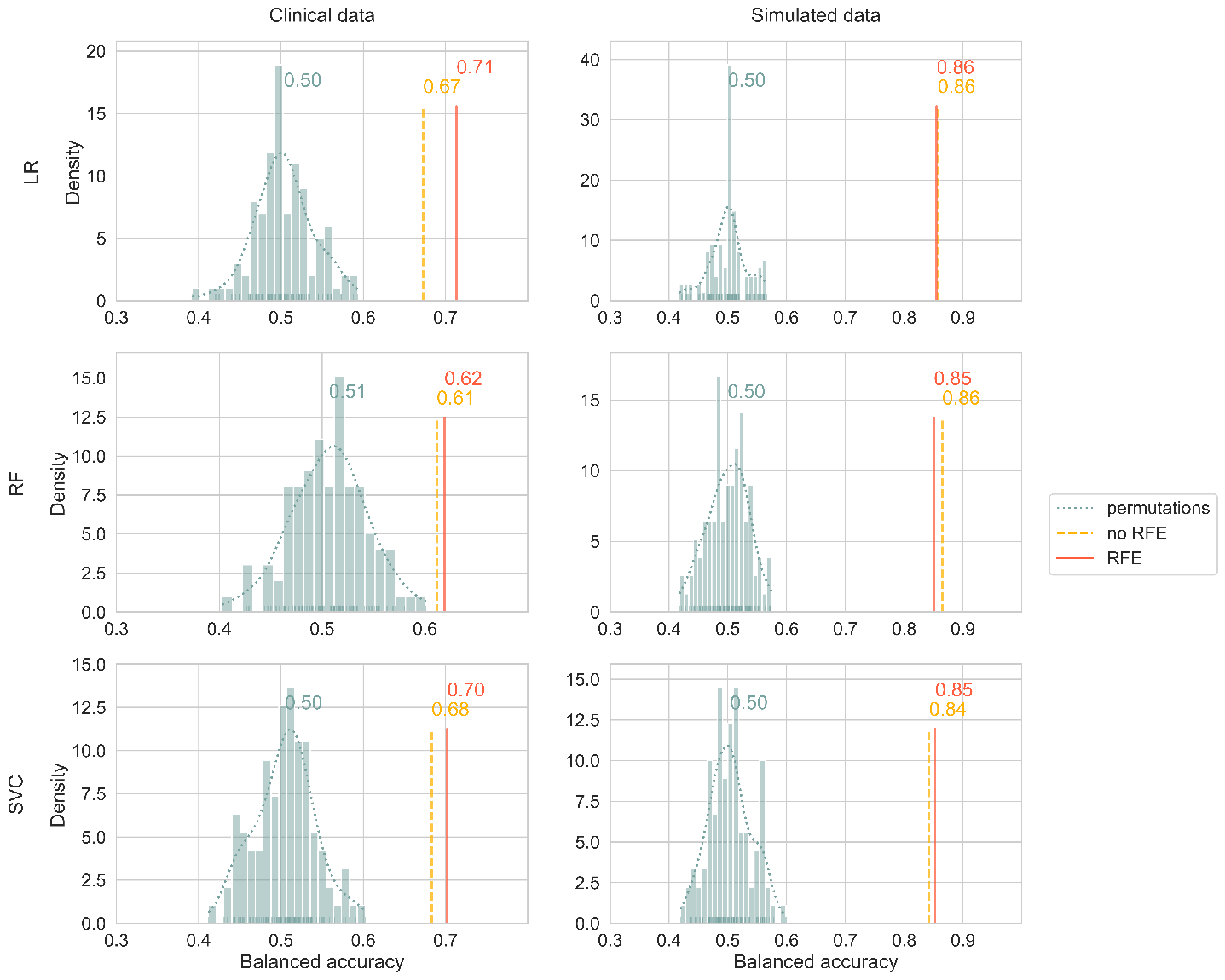


LR, logistic regression; RF, random forest classifier; RFE, recursive feature elimination; SVC, support vector classifier.

### Fig. S3. Receiver operating characteristic curves and corresponding AUC values for all non-permuted models (with and without RFE) across the three classifiers and the two data sets on the validation data.


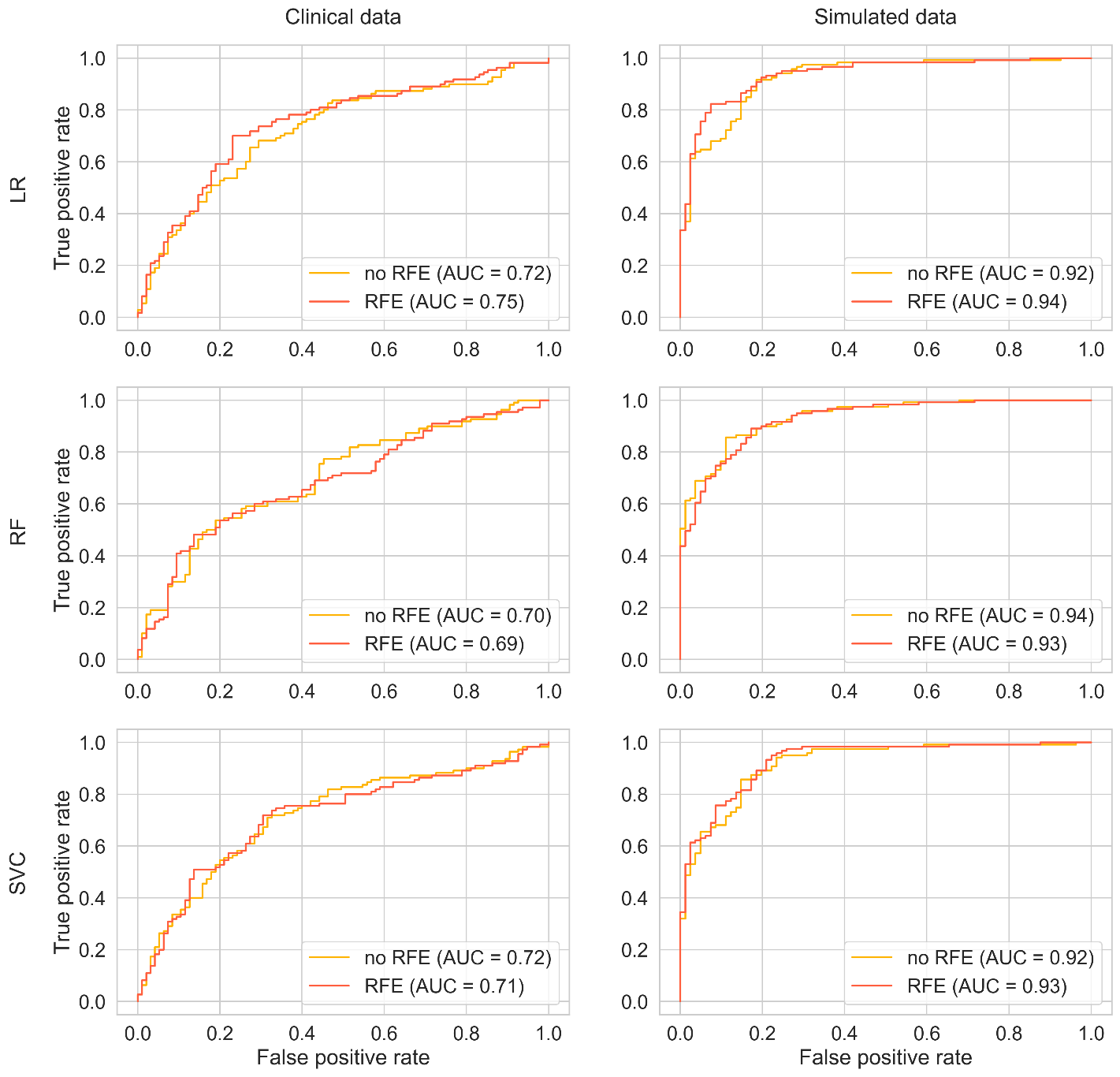


AUC, area under the receiver operating characteristic curve; LR, logistic regression; RF, random forest classifier; RFE, recursive feature elimination; SVC, support vector classifier.

### Fig. S4. Quantile-quantile plots for the 100 permutation runs of each classifier and data set. Empirical distributions of the MCC values on the y-axis are plotted against the theoretical distribution (t(n-2)-distribution) on the x-axis. The dotted line represents the identity function.


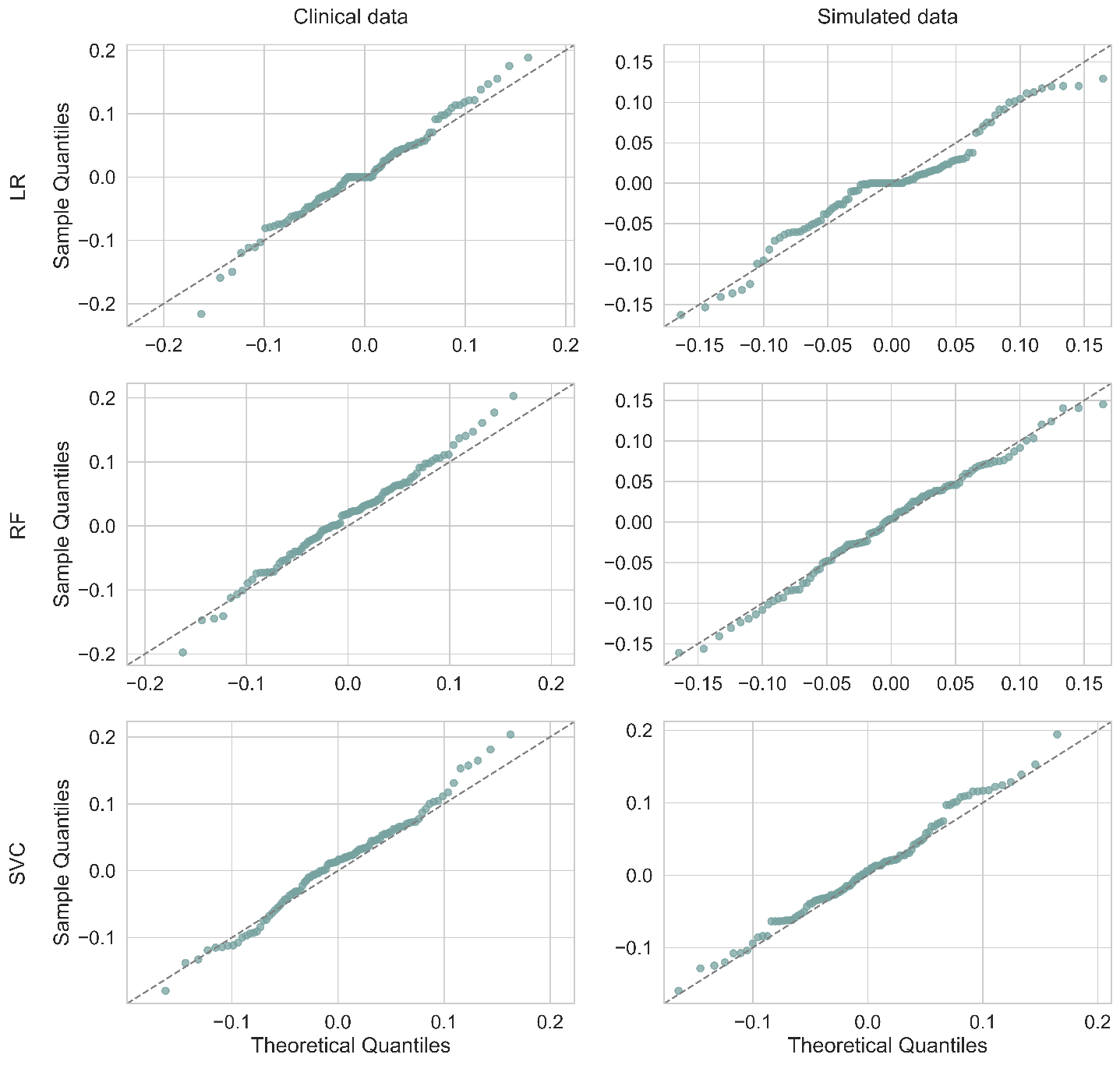


LR, logistic regression; MCC, Matthews correlation coefficient; RF, random forest classifier; SVC, support vector classifier.

### Fig. S5. Permutation importance from 25 permutations for all 113 clinical features, ordered alphabetically and grouped by classifier and model (with and without RFE). The scores show the average decrease in model performance on the validation data when a feature was randomly permuted. Error bars represent 95% confidence intervals. Missing values indicate that a feature was removed by the RFE.


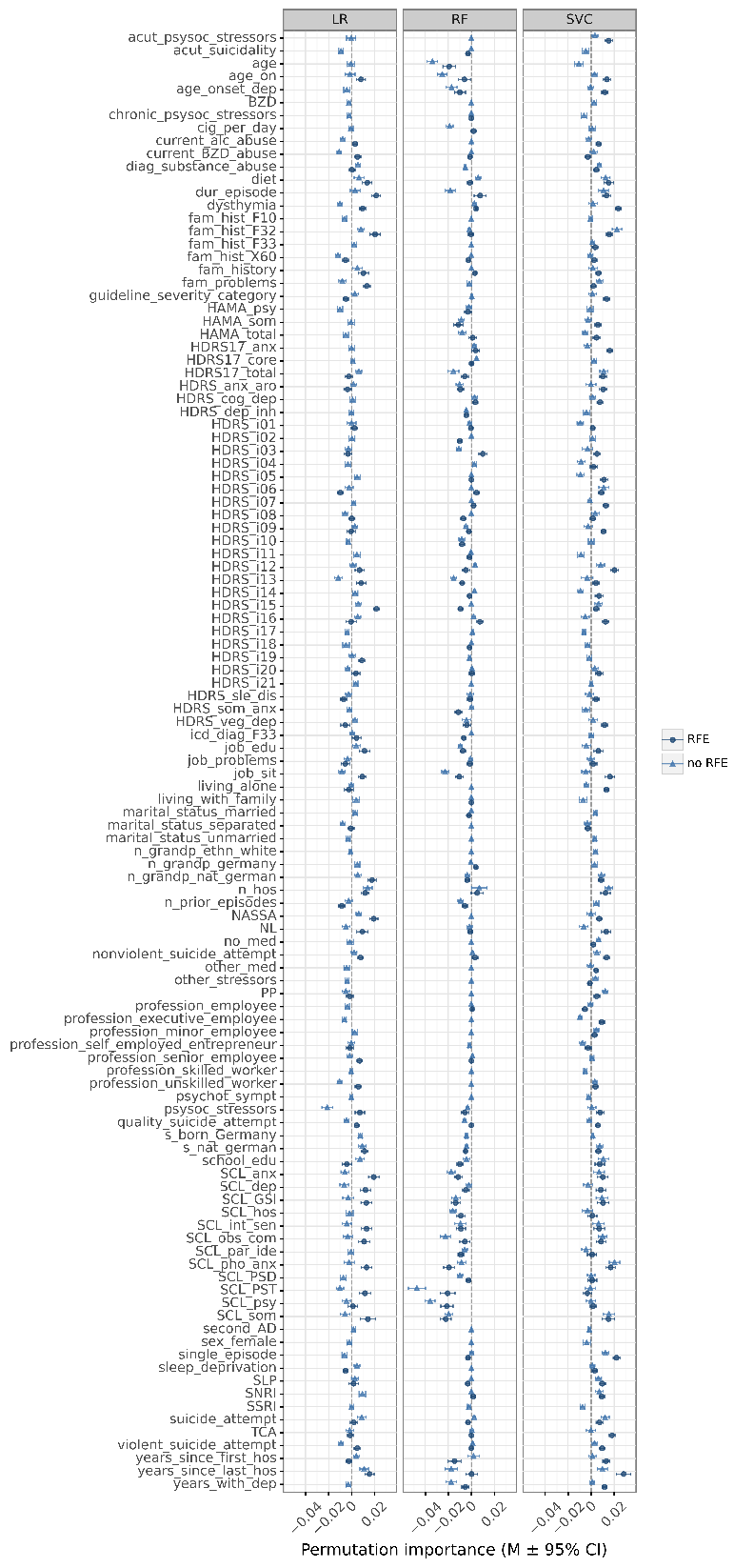


LR, logistic regression; RF, random forest classifier; RFE, recursive feature elimination; SVC, support vector classifier.

### Fig. S6. Permutation importance from 25 permutations for the most informative features from the simulated data set, grouped by classifier and models with and without RFE. Only features that were selected by all 6 models and showed a positive mean importance score (averaged over all 6 models) are presented. The scores show the average decrease in model performance on the validation data when a feature was randomly permuted. Error bars represent 95% confidence intervals.


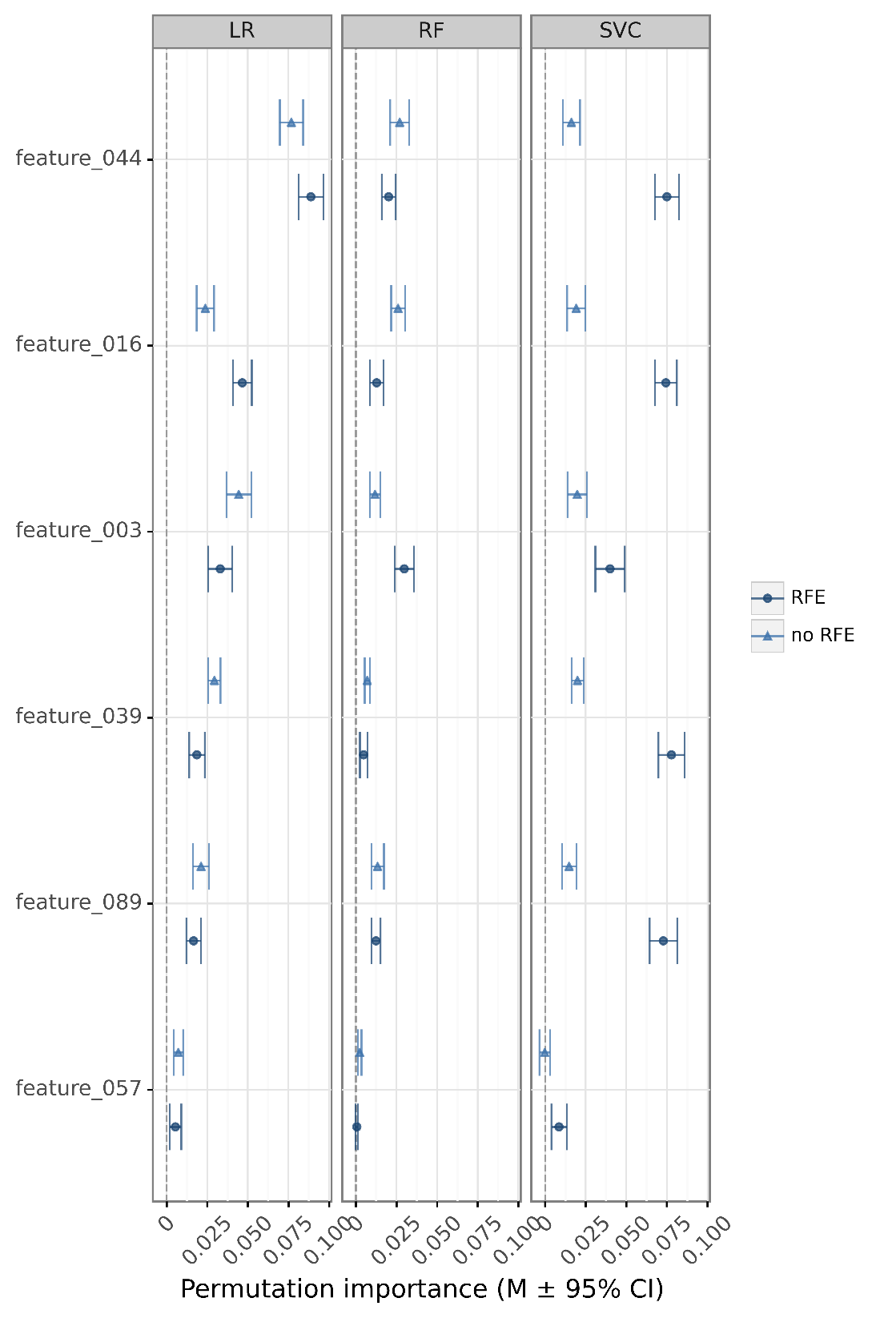


LR, logistic regression; RF, random forest classifier; RFE, recursive feature elimination; SVC, support vector classifier.

### Fig. S7. Permutation importance from 25 permutations for all 125 features from the simulated data set, ordered by number and grouped by classifier and model (with and without RFE). The scores show the average decrease in model performance on the validation data when a feature was randomly permuted. Error bars represent 95% confidence intervals. Missing values indicate that a feature was removed by the RFE.


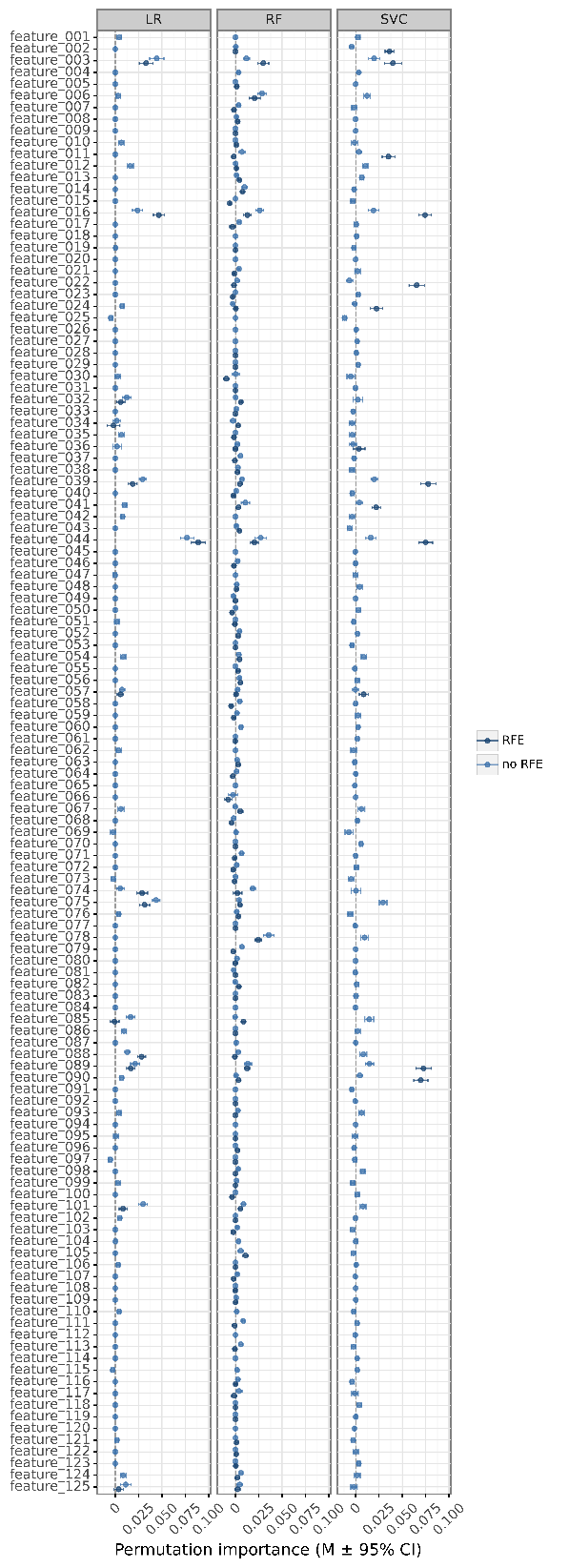


LR, logistic regression; RF, random forest classifier; RFE, recursive feature elimination; SVC, support vector classifier.

# Tables

### Table S1. Baseline features used for predictive modeling in the clinical data set in alphabetical order. After preprocessing, 113 features remained. The table below provides further information on the variables and their distributions.

| **Variable name** | **Variable description** | **Number of missing values (%)** | **Range** | **Comments** |
| --- | --- | --- | --- | --- |
| acut_psysoc_stressors | acute stressors | 0 (0) | 0-1 | 0 = no, 1 = yes |
| acut_suicidality | suicidality prior to admission | 21 (2.05) | 0-1 | 0 = no, 1 = yes |
| age | age at study inclusion | 0 (%) | 18-87 |  |
| age_on | age at onset of any psychiatric disorder | 36 (3.52) | 4-85 |  |
| age_onset_dep | age at onset of depressive disorder | 54 (5.28) | 6.41-85.42 |  |
| BZD | currently taking benzodiacepines | 17 (1.66) | 0-1 | 0 = no, 1 = yes |
| chronic_psysoc_stressors | chroic stressors | 0 (0) | 0-1 | 0 = no, 1 = yes |
| cig_per_day | number of cigarettes smoked per day | 136 (13.31) | 0-60 |  |
| current_alc_abuse | current alcohol abuse | 286 (27.98) | 0-1 | 0 = no, 1 = yes |
| current_BZD_abuse | current benzodiazepine abuse | 288 (28.18) | 0-1 | 0 = no, 1 = yes |
| diag_substance_abuse | additional diagnosis of substance abuse | 0 (0) | 0-1 | 0 = no, 1 = yes |
| diet | whether patient is put on a diet | 240 (23.48) | 0-1 | 0 = no, 1 = yes |
| dur_episode | duration of current episode (weeks) | 97 (9.49) | 0-550 |  |
| dysthymia | preexisting dysthymia | 202 (19.77) | 0-1 | 0 = no, 1 = yes |
| fam_hist_F10 | family history of addiction | 7 (0.68) | 0-1 | 0 = no, 1 = yes |
| fam_hist_F32 | family history of depressive episodes | 6 (0.59) | 0-1 | 0 = no, 1 = yes |
| fam_hist_F33 | family history of recurrent depressive disorder | 6 (0.59) | 0-1 | 0 = no, 1 = yes |
| fam_hist_X60 | family history of suicide | 8 (0.78) | 0-1 | 0 = no, 1 = yes |
| fam_history | psychiatric family history | 16 (1.57) | 0-1 | 0 = none, 1 = positive |
| fam_problems | family-related stressors | 0 (0) | 0-1 | 0 = no, 1 = yes |
| guideline_severity_category | category of depressive symptom severity based on HDRS-17 sum scores according to German treatment guidelines | 7 (0.68) | 1-3 | ordinal scale: 0 = no depression / no clinically remarkable findings or remitted (HDRS-17 ≤ 8), 1 = mild depression (HDRS-17 9-16), 2 = moderate depression (HDRS-17 17-24), 3 = severe depression (HDRS-17 ≥ 25) |
| HAMA_psy | HAMA: psychic anxiety subscale | 206 (20.16) | 1-27 |  |
| HAMA_som | HAMA: somatic anxiety subscale | 206 (20.16) | 0-25 |  |
| HDRS_anx_aro | HDRS: anxious agitation subscale | 125 (12.23) | 0-12 | Ref. (4) |
| HDRS_cog_dep | HDRS: cognitive depression subscale | 129 (12.62) | 0.33-2.83 | Ref. (5) |
| HDRS_dep_inh | HDRS: depressive inhibition subscale | 126 (12.33) | 3-13 | Ref. (4) |
| HDRS_i01 | HDRS item 01: depressed mood | 126 (12.33) | 0-4 |  |
| HDRS_i02 | HDRS item 02: feeling of guilt | 125 (12.23) | 0-4 |  |
| HDRS_i03 | HDRS item 03: suicide | 125 (12.23) | 0-4 |  |
| HDRS_i04 | HDRS item 04: insomnia: early in the night | 126 (12.33) | 0-2 |  |
| HDRS_i05 | HDRS item 05: insomnia: middle of the night | 126 (12.33) | 0-2 |  |
| HDRS_i06 | HDRS item 06: insomnia: early hours of the morning | 127 (12.43) | 0-2 |  |
| HDRS_i07 | HDRS item 07: work and activities | 128 (12.52) | 0-4 |  |
| HDRS_i08 | HDRS item 08: retardation | 126 (12.33) | 0-4 |  |
| HDRS_i09 | HDRS item 09: agitation | 127 (12.43) | 0-4 |  |
| HDRS_i10 | HDRS item 10: anxiety psychic | 126 (12.33) | 0-4 |  |
| HDRS_i11 | HDRS item 11: anxiety somatic (physiological concomitants of anxiety) | 127 (12.43) | 0-4 |  |
| HDRS_i12 | HDRS item 12: somatic symptoms gastro-intestinal | 126 (12.33) | 0-2 |  |
| HDRS_i13 | HDRS item 13: general somatic symptoms | 128 (12.52) | 0-2 |  |
| HDRS_i14 | HDRS item 14: genital symptoms (symptoms such as loss of libido, menstrual disturbances) | 127 (12.43) | 0-2 |  |
| HDRS_i15 | HDRS item 15: hypochondriasis | 126 (12.33) | 0-4 |  |
| HDRS_i16 | HDRS item 16: loss of weight | 125 (12.23) | 0-2 |  |
| HDRS_i17 | HDRS item 17: insight | 125 (12.23) | 0-2 |  |
| HDRS_i18 | HDRS item 18: diurnal variation | 126 (12.33) | 0-2 |  |
| HDRS_i19 | HDRS item 19: depersonalization / derealization | 125 (12.23) | 0-3 |  |
| HDRS_i20 | HDRS item 20: paranoid symptoms | 125 (12.23) | 0-3 |  |
| HDRS_i21 | HDRS item 21: observational/compulsive symptoms | 125 (12.23) | 0-2 |  |
| HDRS_sle_dis | HDRS: sleep disturbances subscale | 126 (12.33) | 0-6 | Ref. (4) |
| HDRS_som_anx | HDRS: somatic anxiety subscale | 126 (12.33) | 0-9 | Ref. (4) |
| HDRS_veg_dep | HDRS: vegetative depression subscale | 132 (12.92) | 0-3.81 | Ref. (5) |
| HDRS17_anx | HDRS anxiety symptom score | 16 (1.57) | 0-8 | sum of items 10 and 11 |
| HDRS17_core | HDRS core symptom score | 19 (1.86) | 2-10 | sum of items 1, 7, and 13 |
| HDRS17_total | HDRS-17: total score | 15 (1.47) | 10-40 |  |
| icd_diag_F33 | ICD-10 diagnosis depressive episode vs. recurrent depressive disorder | 0 (0) | 0-1 | 0 = F32, 1 = F33 |
| job_edu | professional education | 43 (4.21) | 0-3 | ordinal scale: 0 = no job eduction, 1 = apprenticeship, 2 = master school, 3 = college/university |
| job_problems | work-related stressors | 0 (0) | 0-1 | 0 = no, 1 = yes |
| job_sit | job situation | 31 (3.03) | 0-12 | ordinal scale: 0 = working full-time - 12 = unemployed |
| living_alone | living situation: living alone | 0 (0) | 0-1 | 0 = no, 1 = yes |
| living_with_family | living situation: living with family | 0 (0) | 0-1 | 0 = no, 1 = yes |
| marital_status_married | marital status: married | 0 (0) | 0-1 | 0 = no, 1 = yes |
| marital_status_separated | marital status: living separated | 0 (0) | 0-1 | 0 = no, 1 = yes |
| marital_status_unmarried | marital status: unmarried | 0 (0) | 0-1 | 0 = no, 1 = yes |
| n_grandp_ethn_white | number of grandparents with ethnicity "white" | 117 (11.45) | 0-4 |  |
| n_grandp_germany | number of grandparents born in Germany | 121 (11.84) | 0-4 |  |
| n_grandp_nat_german | number of native German speaking grandparents | 120 (11.74) | 0-4 |  |
| n_hos | number of prior hospitalizations | 78 (7.63) | 0-30 |  |
| n_prior_episodes | number of prior depressive episodes | 158 (15.46) | 0-72 |  |
| NASSA | currently taking noradrenergic and specific serotonergic antidepressants | 18 (1.76) | 0-1 | 0 = no, 1 = yes |
| NL | currently taking antipsychotics | 17 (1.66) | 0-1 | 0 = no, 1 = yes |
| no_med | currently not taking any antidepressant medication | 17 (1.66) | 0-1 | 0 = no, 1 = yes |
| nonviolent_suicide_attempt | nonviolent suicide attempts in medical history | 266 (26.03) | 0-1 | 0 = no, 1 = yes |
| other_med | currently taking monoamine oxidase inhibitors or other antidepressants | 17 (1.66) | 0-1 | 0 = no, 1 = yes |
| other_stressors | other stressors | 0 (0) | 0-1 | 0 = no, 1 = yes |
| PP | currently taking mood stabilizers | 17 (1.66) | 0-1 | 0 = no, 1 = yes |
| profession_employee | profession: employee | 0 (0) | 0-1 | 0 = no, 1 = yes |
| profession_executive_employee | profession: executive employee | 0 (0) | 0-1 | 0 = no, 1 = yes |
| profession_minor_employee | profession: minor employee | 0 (0) | 0-1 | 0 = no, 1 = yes |
| profession_self_employed_entrepreneur | profession: self-employed entrepreneur | 0 (0) | 0-1 | 0 = no, 1 = yes |
| profession_senior_employee | profession: senior employee | 0 (0) | 0-1 | 0 = no, 1 = yes |
| profession_skilled_worker | profession: skilled worker | 0 (0) | 0-1 | 0 = no, 1 = yes |
| profession_unskilled_worker | profession: unskilled worker | 0 (0) | 0-1 | 0 = no, 1 = yes |
| psychot_sympt | presence of psychotic symptoms | 29 (2.84) | 0-1 | 0 = no, 1 = yes |
| psysoc_stressors | psychosocial stressors prior to admission | 0 (0) | 0-1 | 0 = no, 1 = yes |
| quality_suicide_attempt | quality of suicide attempt prior to admission | 76 (7.44) | 0-3 | ordinal scale: 0 = none, 1 = self-harm without suicide intention, 2 = other suicide attempts, 3 = clearly dangerous suicide attempt |
| s_born_Germany | whether patient was born in Germany | 71 (6.95) | 0-1 | 0 = no, 1 = yes |
| s_nat_german | native German speaker | 71 (6.95) | 0-1 | 0 = no, 1 = yes |
| school_edu | school education | 45 (4.40) | 0-7 | ordinal scale: 0 = left school without certificate - 7 = high-school diploma |
| SCL_anx | SCL-90-R: anxiety | 260 (25.44) | 0-3.7 |  |
| SCL_dep | SCL-90-R: depression | 258 (25.24) | 0-3.85 |  |
| SCL_GSI | SCL-90-R: global severity | 261 (25.54) | 0.09-3.33 |  |
| SCL_hos | SCL-90-R: hostility | 263 (25.73) | 0-3.67 |  |
| SCL_int_sen | SCL-90-R: interpersonal sensitivity | 260 (25.44) | 0-4 |  |
| SCL_obs_com | SCL-90-R: obsessive-compulsive | 261 (25.54) | 0-4 |  |
| SCL_par_ide | SCL-90-R: paranoid ideation | 261 (25.54) | 0-3.5 |  |
| SCL_pho_anx | SCL-90-R: phobic anxiety | 261 (25.54) | 0-4 |  |
| SCL_PSD | SCL-90-R: positive symptom distress | 117 (11.45) | 0-3.76 |  |
| SCL_PST | SCL-90-R: positive symptom total | 111 (10.86) | 0-90 |  |
| SCL_psy | SCL-90-R: psychoticism | 263 (25.73) | 0-3.8 |  |
| SCL_som | SCL-90-R: somatization | 259 (25.34)) | 0-3.83 |  |
| second_AD | currently taking antidepressants coming from at least two different classes | 32 (3.13) | 0-1 | 0 = no, 1 = yes |
| sex_female | sex | 0 (%) | 0-1 | 0 = male, 1 = female |
| single_episode | single depressive episode | 65 (6.36) | 0-1 | 0 = no, 1 = yes |
| sleep_deprivation | method of sleep deprivation | 158 (15.46) | 0-2 | ordinal scale: 0 = none, 1 = partial, 2 = total |
| SLP | currently taking sleep medication | 17 (1.66) | 0-1 | 0 = no, 1 = yes |
| SNRI | currently taking serotonin-norepinephrnie reuptake inhibitors | 17 (1.66) | 0-1 | 0 = no, 1 = yes |
| SSRI | currently taking selective serotonin reuptake inhibitors | 17 (1.66) | 0-1 | 0 = no, 1 = yes |
| suicide_attempt | suicide attempt | 91 (8.90) | 0-1 | 0 = no, 1 = yes |
| TCA | currently taking tricyclic antidepressants | 18 (1.76) | 0-1 | 0 = no, 1 = yes |
| violent_suicide_attempt | violent suicide attempts in medical history | 281 (27.50 | 0-1 | 0 = no, 1 = yes |
| years_since_first_hos | time since first hospitalization (years) | 39 (3.82) | 0-54.10 |  |
| years_since_last_hos | time since last hospitalization (years) | 95 (9.30) | 0-86.57 |  |
| years_with_dep | time since diagnosis of depressive disorder (years) | 56 (5.48) | 0-66.94 |  |

HAMA, Hamilton Anxiety Rating Scale (6); HDRS, Hamilton Rating Scale for Depression (1); HDRS-17, 17-item version of the HDRS; ICD-10, International Classification of Diseases (2); SCL-90-R, Symptom Checklist-90-revised (7).

### Table S2. TRIPOD Checklist for Prediction Model Development and Validation (8)

| **Section/Topic** | **Item** |  | **Checklist Item** | **Page** |
| --- | --- | --- | --- | --- |
| **Title and abstract** | | | | |
| Title | 1 | D;V | Identify the study as developing and/or validating a multivariable prediction model, the target population, and the outcome to be predicted. | 1 |
| Abstract | 2 | D;V | Provide a summary of objectives, study design, setting, participants, sample size, predictors, outcome, statistical analysis, results, and conclusions. | 1 |
| **Introduction** | | | | |
| Background and objectives | 3a | D;V | Explain the medical context (including whether diagnostic or prognostic) and rationale for developing or validating the multivariable prediction model, including references to existing models. | 1-3 |
|  | 3b | D;V | Specify the objectives, including whether the study describes the development or validation of the model or both. | 3 |
| **Methods** | | | | |
| Source of data | 4a | D;V | Describe the study design or source of data (e.g., randomized trial, cohort, or registry data), separately for the development and validation data sets, if applicable. | 3 |
|  | 4b | D;V | Specify the key study dates, including start of accrual; end of accrual; and, if applicable, end of follow-up. | Ref.^19^ |
| Participants | 5a | D;V | Specify key elements of the study setting (e.g., primary care, secondary care, general population) including number and location of centres. | Ref.^19^ |
|  | 5b | D;V | Describe eligibility criteria for participants. | 3 |
|  | 5c | D;V | Give details of treatments received, if relevant. | Ref.^19^ |
| Outcome | 6a | D;V | Clearly define the outcome that is predicted by the prediction model, including how and when assessed. | 3 |
|  | 6b | D;V | Report any actions to blind assessment of the outcome to be predicted. | - |
| Predictors | 7a | D;V | Clearly define all predictors used in developing or validating the multivariable prediction model, including how and when they were measured. | 3-4; Table S1 |
|  | 7b | D;V | Report any actions to blind assessment of predictors for the outcome and other predictors. | - |
| Sample size | 8 | D;V | Explain how the study size was arrived at. | 3;  Fig. S1 |
| Missing data | 9 | D;V | Describe how missing data were handled (e.g., complete-case analysis, single imputation, multiple imputation) with details of any imputation method. | 5 |
| Statistical analysis methods | 10a | D | Describe how predictors were handled in the analyses. | 3-5; Fig. S1 |
|  | 10b | D | Specify type of model, all model-building procedures (including any predictor selection), and method for internal validation. | 4-5 |
|  | 10c | V | For validation, describe how the predictions were calculated. | 5 |
|  | 10d | D;V | Specify all measures used to assess model performance and, if relevant, to compare multiple models. | 5 |
|  | 10e | V | Describe any model updating (e.g., recalibration) arising from the validation, if done. | - |
| Risk groups | 11 | D;V | Provide details on how risk groups were created, if done. | - |
| Development vs. validation | 12 | V | For validation, identify any differences from the development data in setting, eligibility criteria, outcome, and predictors. | - |
| **Results** | | | | |
| Participants | 13a | D;V | Describe the flow of participants through the study, including the number of participants with and without the outcome and, if applicable, a summary of the follow-up time. A diagram may be helpful. | 5 |
|  | 13b | D;V | Describe the characteristics of the participants (basic demographics, clinical features, available predictors), including the number of participants with missing data for predictors and outcome. | 5 (Table 2); Table S1 |
|  | 13c | V | For validation, show a comparison with the development data of the distribution of important variables (demographics, predictors and outcome). | 5 (Table 2) |
| Model development | 14a | D | Specify the number of participants and outcome events in each analysis. | 5 |
|  | 14b | D | If done, report the unadjusted association between each candidate predictor and outcome. | - |
| Model specification | 15a | D | Present the full prediction model to allow predictions for individuals (i.e., all regression coefficients, and model intercept or baseline survival at a given time point). | - |
|  | 15b | D | Explain how to the use the prediction model. | - |
| Model performance | 16 | D;V | Report performance measures (with CIs) for the prediction model. | 5-6; Fig. S3; Table S4 |
| Model-updating | 17 | V | If done, report the results from any model updating (i.e., model specification, model performance). | - |
| **Discussion** | | | | |
| Limitations | 18 | D;V | Discuss any limitations of the study (such as nonrepresentative sample, few events per predictor, missing data). | 10-11 |
| Interpretation | 19a | V | For validation, discuss the results with reference to performance in the development data, and any other validation data. | 10 |
|  | 19b | D;V | Give an overall interpretation of the results, considering objectives, limitations, results from similar studies, and other relevant evidence. | 8-11 |
| Implications | 20 | D;V | Discuss the potential clinical use of the model and implications for future research. | 8-11 |
| **Other information** | | | | |
| Supplementary information | 21 | D;V | Provide information about the availability of supplementary resources, such as study protocol, Web calculator, and data sets. | Suppl. Material |
| Funding | 22 | D;V | Give the source of funding and the role of the funders for the present study. | 11 |

Note: Items relevant only to the development of a prediction model are denoted by D, items relating solely to a validation of a prediction model are denoted by V, and items relating to both are denoted D;V. We recommend using the TRIPOD Checklist in conjunction with the TRIPOD Explanation and Elaboration document.

### Table S3. Matthews correlation coefficients and corresponding p-values for non-permuted models (with and without recursive feature elimination). P-values were derived from the theoretical null distribution (t(n-2)-distribution).

|  |  | RFE | | no RFE | |
| --- | --- | --- | --- | --- | --- |
|  |  | MCC | p | MCC | p |
| Clinical data | LR | 0.425 | 2.09 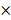 10^-10^ | 0.350 | 2.76 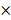 10^-7^ |
|  | RF | 0.237 | 6.13 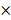 10^-4^ | 0.224 | 1.27 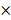 10^-3^ |
|  | SVC | 0.403 | 2.06 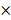 10^-9^ | 0.365 | 7.31 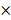 10^-8^ |
| Simulated data | LR | 0.718 | 8.58 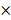 10^-34^ | 0.719 | 7.40 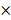 10^-34^ |
|  | RF | 0.700 | 1.77 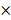 10^-31^ | 0.724 | 1.60 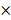 10^-34^ |
|  | SVC | 0.709 | 1.46 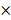 10^-32^ | 0.688 | 4.78 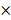 10^-30^ |

LR, elastic-net regularized logistic regression; MCC, Matthews correlation coefficient; RF, random forest classifier; RFE, recursive feature elimination; SVC, support vector classifier.

### Table S4. Confusion matrices and derived performance metrics including 95% confidence intervals for all non-permuted models on the validation data.

|  |  | **Clinical data** | | | | **Simulated data** | | | |
| --- | --- | --- | --- | --- | --- | --- | --- | --- | --- |
| **LR** | no RFE |  |  | Predicted response | |  |  | Predicted label | |
|  |  |  |  | no | yes |  |  | 0 | 1 |
|  |  | True response | no | 57 | 38 | True label | 0 | 66 | 15 |
|  |  |  | yes | 28 | 82 |  | 1 | 12 | 107 |
|  |  | Sens | 0.75 [0.69, 0.81] | | | Sens | 0.90 [0.86, 0.94] | | |
|  |  | Spec | 0.60 [0.53, 0.67] | | | Spec | 0.82 [0.77, 0.87] | | |
|  |  | PPV | 0.68 [0.62, 0.74] | | | PPV | 0.88 [0.83, 0.93] | | |
|  |  | NPV | 0.67 [0.61, 0.73] | | | NPV | 0.85 [0.80, 0.90] | | |
|  |  | PLR | 1.86 [1.42, 2.44] | | | PLR | 4.86 [3.06, 7.70] | | |
|  |  | NLR | 0.42 [0.30, 0.61] | | | NLR | 0.12 [0.07, 0.21] | | |
|  | RFE |  |  | Predicted Response | |  |  | Predicted label | |
|  |  |  |  | no | yes |  |  | 0 | 1 |
|  |  | True Response | no | 69 | 26 | True label | 0 | 65 | 16 |
|  |  |  | yes | 33 | 77 |  | 1 | 11 | 108 |
|  |  | Sens | 0.70 [0.64, 0.76] | | | Sens | 0.91 [0.87, 0.95] | | |
|  |  | Spec | 0.73 [0.67, 0.79] | | | Spec | 0.80 [0.74, 0.86] | | |
|  |  | PPV | 0.75 [0.69, 0.81] | | | PPV | 0.87 [0.82, 0.92] | | |
|  |  | NPV | 0.68 [0.62, 0.74] | | | NPV | 0.86 [0.81, 0.91] | | |
|  |  | PLR | 2.56 [1.80, 3.63] | | | PLR | 4.59 [2.95, 7.15] | | |
|  |  | NLR | 0.41 [0.30, 0.56] | | | NLR | 0.12 [0.06, 0.20] | | |
| **RF** | no RFE |  |  | Predicted response | |  |  | Predicted label | |
|  |  |  |  | no | yes |  |  | 0 | 1 |
|  |  | True response | no | 54 | 41 | True label | 0 | 70 | 11 |
|  |  |  | yes | 38 | 72 |  | 1 | 16 | 103 |
|  |  | Sens | 0.66 [0.60, 0.72] | | | Sens | 0.87 [0.82, 0.92] | | |
|  |  | Spec | 0.57 [0.50, 0.64] | | | Spec | 0.86 [0.81, 0.91] | | |
|  |  | PPV | 0.64 [0.57, 0.71] | | | PPV | 0.90 [0.86, 0.94] | | |
|  |  | NPV | 0.59 [0.52, 0.66] | | | NPV | 0.81 [0.76, 0.86] | | |
|  |  | PLR | 1.52 [1.16, 1.98] | | | PLR | 6.37 [3.66, 11.09] | | |
|  |  | NLR | 0.61 [0.45, 0.83] | | | NLR | 0.16 [0.10, 0.25] | | |
|  | RFE |  |  | Predicted Response | |  |  | Predicted label | |
|  |  |  |  | no | yes |  |  | 0 | 1 |
|  |  | True Response | no | 58 | 37 | True label | 0 | 67 | 14 |
|  |  |  | yes | 41 | 69 |  | 1 | 15 | 104 |
|  |  | Sens | 0.63 [0.56, 0.70] | | | Sens | 0.87 [0.82, 0.92] | | |
|  |  | Spec | 0.61 [0.54, 0.68] | | | Spec | 0.83 [0.78, 0.88] | | |
|  |  | PPV | 0.65 [0.58, 0.72] | | | PPV | 0.88 [0.83, 0.93] | | |
|  |  | NPV | 0.59 [0.52, 0.66] | | | NPV | 0.82 [0.77, 0.87] | | |
|  |  | PLR | 1.61 [1.21, 2.15] | | | PLR | 5.06 [3.12, 8.18] | | |
|  |  | NLR | 0.61 [0.46, 0.82] | | | NLR | 0.15 [0.09, 0.25] | | |
| **SVC** | no RFE |  |  | Predicted response | |  |  | Predicted label | |
|  |  |  |  | no | yes |  | 0  1 | 0 | 1 |
|  |  | True response | no | 65 | 30 | True label |  | 65 | 16 |
|  |  |  | yes | 35 | 75 |  |  | 14 | 105 |
|  |  | Sens | 0.68 [0.62, 0.74] | | | Sens | 0.88 [0.83, 0.93] | | |
|  |  | Spec | 0.68 [0.62, 0.74] | | | Spec | 0.80 [0.74, 0.86] | | |
|  |  | PPV | 0.71 [0.65, 0.77] | | | PPV | 0.87 [0.82, 0.92] | | |
|  |  | NPV | 0.65 [0.58, 0.72] | | | NPV | 0.82 [0.77, 0.87] | | |
|  |  | PLR | 2.16 [1.56, 2.98] | | | PLR | 4.47 [2.87, 6.96] | | |
|  |  | NLR | 0.47 [0.34, 0.63] | | | NLR | 0.15 [0.09, 0.24] | | |
|  | RFE |  |  | Predicted Response | |  |  | Predicted label | |
|  |  |  |  | no | yes |  | 0  1 | 0 | 1 |
|  |  | True Response | no | 66 | 29 | True label |  | 66 | 15 |
|  |  |  | yes | 32 | 78 |  |  | 13 | 106 |
|  |  | Sens | 0.71 [0.65, 0.77] | | | Sens | 0.89 [0.84, 0.94] | | |
|  |  | Spec | 0.70 [0.64, 0.76] | | | Spec | 0.82 [0.77, 0.87] | | |
|  |  | PPV | 0.73 [0.67, 0.79] | | | PPV | 0.88 [0.83, 0.93] | | |
|  |  | NPV | 0.67 [0.61, 0.73] | | | NPV | 0.84 [0.79, 0.89] | | |
|  |  | PLR | 2.32 [1.68, 3.22] | | | PLR | 4.81 [3.03, 7.63] | | |
|  |  | NLR | 0.42 [0.30, 0.58] | | | NLR | 0.13 [0.08, 0.23] | | |

LR, elastic-net regularized logistic regression; NPV, negative predictive value; PPV, positive predictive value; RF, random forest classifier; RFE, recursive feature elimination; Sens, sensitivity; Spec, specificity; SVC, support vector classifier.

### Table S5. Results from Kolmogorov-Smirnov tests comparing the empirical MCC distributions of the permutation runs to the theoretical null distribution (t(n-2)-distribution).

|  |  | D | p |
| --- | --- | --- | --- |
| Clinical data | LR | 0.10 | 0.253 |
|  | RF | 0.13 | 0.067 |
|  | SVC | 0.11 | 0.147 |
| Simulated data | LR | 0.13 | 0.065 |
|  | RF | 0.05 | 0.958 |
|  | SVC | 0.08 | 0.459 |

D, maximal absolute difference; LR, elastic-net regularized logistic regression; RF, random forest classifier; SVC, support vector classifier.

# References

1. Hamilton M. A rating scale for depression. J Neurol Neurosurg Psychiatry. 1960;23(1):56–62.

2. World Health Organization. The ICD-10 classification of mental and behavioural disorders: Clinical descriptions and diagnostic guidelines. Geneva: World Health Organisation; 1992.

3. Hennings JM, Owashi T, Binder EB, Horstmann S, Menke A, Kloiber S, et al. Clinical characteristics and treatment outcome in a representative sample of depressed inpatients - Findings from the Munich Antidepressant Response Signature (MARS) project. J Psychiatr Res. 2009;43(3):215–29.

4. Maier W, Philipp M, Gerken A. Dimensionen der Hamilton-Depressionsskala (HAMD). Eur Arch Psychiatry Neurol Sci. 1985;234(6):417–22.

5. Rhoades HM, Overall JE. The Hamilton Depression Scale: Factor Scoring and Profile. Psychopharmacol Bull [Internet]. 1983;140(3):91–6. Available from: https://books.google.de/books?hl=de&lr=&id=bpDDYcIIAi8C&oi=fnd&pg=PA91&dq=hamilton+depression+overall+rhoades&ots=gCum7TZpzj&sig=gkphUlj59ADQxZj6ICeH8Qz38Iw#v=onepage&q=hamilton depression overall rhoades&f=false

6. Hamilton M. The assessment of anxiety states by rating. Br J Med Psychol. 1959;50–5.

7. Derogatis LR. SCL-90-R : administration, scoring and procedures. Man II R Version Other Instruments Psychopathol Rat Scale Ser [Internet]. 1983 [cited 2021 Sep 8]; Available from: http://ci.nii.ac.jp/naid/10019227338/en/

8. Collins GS, Reitsma JB, Altman DG, Moons KGM. Transparent Reporting of a multivariable prediction model for Individual Prognosis Or Diagnosis (TRIPOD): The TRIPOD Statement. Br J Surg. 2015;102(3):148–58.
